# A Smart Nanomedicine Unleashes a Dual Assault of Glucose Starvation and Cuproptosis to Supercharge αPD‐L1 Therapy

**DOI:** 10.1002/advs.202411378

**Published:** 2024-12-04

**Authors:** Yiming Xu, Yuan Wu, Xinjie Zheng, Dongxue Wang, Hangqi Ni, Weiyu Chen, Kai Wang

**Affiliations:** ^1^ Department of Respiratory and Critical Care Medicine Center for Oncology Medicine The Fourth Affiliated Hospital of School of Medicine and International School of Medicine International Institutes of Medicine Zhejiang University Yiwu 322000 China; ^2^ Zhejiang Key Laboratory of Precision Diagnosis and Treatment for Lung Cancer Yiwu 322000 China; ^3^ College of Jiyang Zhejiang A&F University Zhuji 311800 China; ^4^ Department of Nuclear Medicine The Second Affiliated Hospital of Harbin Medical University Harbin 150000 China

**Keywords:** biomimetic nanomedicine, cuproptosis, glucose metabolisms, lung cancer, PD‐L1

## Abstract

Combination therapy has become a promising strategy for promoting the outcomes of anti‐programmed death ligand‐1 (αPD‐L1) therapy in lung cancer. Among all, emerging strategies targeting cancer metabolism have shown great potency in treating cancers with immunotherapy. Here, alteration in glucose and copper metabolisms is found to synergistically regulate PD‐L1 expression in lung cancer cells. Thus, an intelligent biomimetic nano‐delivery system is synthesized by camouflaging lung cancer cell membranes onto glucose oxidase‐loaded Cu‐LDHs (CMGCL) for cancer metabolism targeted interference. Such novel nanomedicine is able to induce lung cancer cell cuproptosis and PD‐L1 upregulation significantly via self‐amplified cascade reactions. Meanwhile, with a decent cancer cell membrane coating, CMGCL exhibited great biosafety, tumor‐targeted efficiency and anti‐tumor effects in LLC lung tumor‐bearing mice models. Additionally, a combination of CMGCL can sensitize the therapeutic effects of αPD‐L1, substantially promoting tumor inhibition in both subcutaneous and lung metastasis LLC‐bearing mice models. Overall, these findings highlight the potential connections between glucose metabolism and cell cuproptosis, offering a promising approach for treating lung cancer by integrating starvation, cuproptosis, and immunotherapy.

## Graphical abstract

An intelligent biomimetic nanomedicine is synthesized by camouflaging lung cancer cell membranes onto glucose oxidase‐loaded Cu‐LDHs (CMGCL) for cancer metabolism targeted interference. The CMGCL can achieve great tumor targeting efficacy and upregulate programmed death ligand‐1 (PD‐L1) expression on LLC cells by simultaneously inducing glucose starvation and cuproptosis, thus effectively sensitizing αPD‐L1 therapy against subcutaneous or lung metastatic LLC tumors.

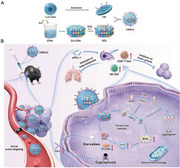

## 1 Introduction

To date, lung cancer still contributes to most cancer‐related deaths worldwide, with ≈2.4 million new diagnoses and 1.3 million fatalities reported annually.^[^ ^1^ ^]^ Recently, immunotherapy, particularly immune checkpoint inhibitors (ICIs), has garnered significant attention due to the desirable therapeutic outcomes for most lung cancer patients.^[^ ^2^ ^]^ For instance, the anti‐programmed death ligand‐1 (αPD‐L1) antibody, as one of the most widely used ICIs, has been approved for more than 20 types of solid tumors, including lung cancer. However, some patients respond poorly or generate resistance due to a shortage of PD‐L1 expression and dysfunctional immune response within the tumor microenvironment (TME).^[^ ^2^, ^3^ ^]^ Moreover, similar to other traditional therapies (e.g., chemotherapy or radiotherapy), the accompanying side effects, such as immune‐related adverse effects (irAEs), also limit the clinical outcomes of immunotherapy. Thus, mono immunotherapy is insufficient for all lung cancer treatments, which may be overcome via effective combinational strategies.

In the clinic, a series of combination therapies have been applied to enhance immunotherapy, such as chemotherapy, antiangiogenics, or radiotherapy.^[^ ^4^ ^]^ However, the inevitable side effects are generally accompanied by these traditional combined therapies. Notably, as an emerging cancer therapeutic approach, cancer metabolism‐targeted therapy has drawn great interest.^[^ ^5^ ^]^ Abnormal metabolism with excessive nutrition, especially glucose addiction, is one of the hallmarks of cancer tissues, offering an excellent opportunity to restrain cancerous cells by limiting glucose consumption.^[^ ^6^ ^]^ Glucose oxidase (GOx), a natural enzyme catalyst, can effectively convert glucose into gluconic acid and hydrogen peroxide (H_2_O_2_),^[^ ^7^ ^]^ thus being an excellent inducer for tumor starvation therapy and a potential regulator for glucose metabolism. More importantly, glucose starvation might affect PD‐L1 expression on cancer cells,^[^ ^8^ ^]^ while blocking PD‐L1 directly on tumors has been reported to decrease tumor glycolytic metabolism and promote T cell functions.^[^ ^9^ ^]^ Thus, it should be an exciting and promising strategy to combine glucose starvation with αPD‐L1 therapy.

In addition to aerobic glycolysis, the tricarboxylic acid (TCA) cycle occurring in mitochondria is another essential biosynthetic metabolic reaction for cancer cell survival, largely dependent on glucose supply.^[^ ^10^ ^]^ Metal ions are integral to forming many enzyme complexes within mitochondria, essential for the TCA cycle. Their balances are crucial for various cellular functions, thus becoming a promising target for cancer therapies,^[^ ^11^ ^]^ such as metal‐dependent cancer cell deaths (oxidative stress caused by metal ion imbalance).^[^ ^12^ ^]^ Notably, intracellular overloading copper (Cu) can directly bind to TCA‐related enzymes and trigger proteotoxic stress, leading to a distinct cell death, cuproptosis.^[^ ^13^ ^]^ An increasing body of research has revealed the therapeutic potential of targeting cuproptosis in cancer treatments.^[^ ^14^ ^]^ Meanwhile, by showing redox capabilities, Cu is able to act as a powerful enhancer for Fenton‐like reactions through glutathione (GSH) depletion and hydroxyl radicals (•OH) generation from H_2_O_2_.^[^ ^15^ ^]^ Moreover, recent studies have demonstrated that intratumoral Cu levels are essential in regulating PD‐L1 expression,^[^ ^16^ ^]^ and cuproptosis might correlate with tumor immune evasion.^[^ ^17^ ^]^ Accordingly, seeking available strategies to combine glucose starvation and cuproptosis might be a reinforcement for lung cancer αPD‐L1 therapy.

Intelligent delivery systems, such as nanomedicine have been applied to regulate cancer metabolism multidimensionally, with much better peripheral clearance, targeting, and biosafety compared with traditional combination therapies.^[^ ^18^ ^]^ Biomimetic nanoparticles camouflaged with cell membranes, especially cancer cell membrane‐coated nanoparticles (CM‐NPs), demonstrate excellent biocompatibility and homologous targeting effect due to specific affinity toward parent cancer cells.^[^ ^19^ ^]^ Meanwhile, layered double hydroxide nanoparticles (LDHs), unique 2D‐structured nanomaterials, exhibit excellent physiochemical features, including biocompatibility, pH‐sensitive biodegradability, and high loading capacity.^[^ ^20^ ^]^ In addition, the flexibility of layer structure enables LDHs to carry various electrical items and metal ions (e.g., Zn^2+^ or Mn^2+^) via ion exchange.^[^ ^21^ ^]^ Green synthesized LDHs without hydrothermal are small in size and suitable for ion substitution. With these desirable capacities, LDHs have been widely explored in biomedical fields (drug delivery, vaccine, and disease theranostics), which also show great potential as the core of CM‐NPs.^[^ ^22^ ^]^

**Scheme 1:**
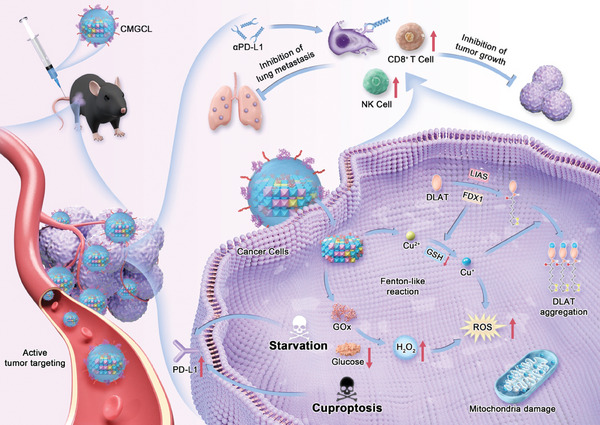
Scheme illustration depicting the sensitization of lung cancer αPD‐L1 therapy by CMGCL‐induced glucose starvation and cuproptosis synergistically.

In this study, we first revealed that glucose limitation exhibited an intrinsic correlation with copper balance, and integrated with cuproptosis could synergistically upregulate PD‐L1 expression on lung cancer cells. Inspired by these findings, a novel biomimetic nanomedicine, LLC lung cancer cell membrane (CM)‐camouflaged copper substituted LDHs with GOx loading (CMGCL) was designed for αPD‐L1 combination therapy. The CMGCL could successfully achieve GSH consumption and glucose limitation, then convert the generated H_2_O_2_ into toxic •OH through a Fenton‐like reaction. These cascade reactions in lung cancer cells exhibited high cytotoxicity through cell cuproptosis both in vitro and in vivo. CM coating LDHs also demonstrated a desirable lung tumor‐targeting efficiency. Moreover, CMGCL could increase PD‐L1 expression on lung cancer cells by simultaneously mediating cancer starvation and cuproptosis, thus effectively sensitizing αPD‐L1 therapy against both primary and metastatic LLC tumors. Overall, the combinational strategy proposed in our work demonstrates the synergistic effect of glucose starvation and cuproptosis on enhancing lung cancer αPD‐L1 therapy, showing the potential of CMGCL as an effective sensitizer for αPD‐L1 combined therapy (**Scheme** **1**).

## 2 Results

### 2.1 PD‐L1 Upregulation Induced by Glucose Starvation and Cuproptosis Synergistically

**Figure 1:**
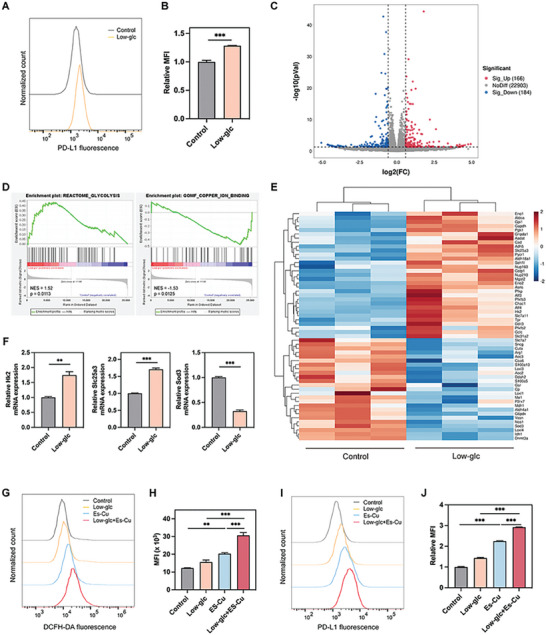
PD‐L1 upregulation induced by glucose starvation and cuproptosis synergistically. A,B) Representative flow cytometry histograms and quantitative analysis of PD‐L1 expression on LLC cells after low‐glc treatment for 24 h. MFI: mean fluorescence intensity. C) Volcano plots displayed differentially expressed genes from RNA‐seq comparing low‐glc with control groups. D) GSEA analysis of glycolysis and copper ion binding pathways in control and low‐glc groups. NES: normalized enrichment score. E) Heatmap analysis of gene expressions between control and low‐glc groups in LLC cells. F) The relative mRNA levels of Hk2, Slc25a3 and Sod3 in different groups were measured by RT‐qPCR and were normalized by the mRNA expression levels of β‐actin. G,H) Flow cytometry histograms and quantitative analysis of DCFH‐DA fluorescence of LLC cells after different treatments. I,J) Representative flow cytometry histograms and quantitative analysis of PD‐L1 expression on LLC cells after different treatments. Data are depicted as mean ± SEM; *n* = 3. ^**^ *p* < 0.01, ^***^ *p* < 0.001.

To evaluate the influence of glucose starvation on PD‐L1 expression of lung cancer cells, LLC cells were cultured in a low‐glucose (low‐glc) medium in contrast to a normal culture medium (control). Notably, the PD‐L1 expression on LLC cells was upregulated significantly after glucose starvation (**Figure** **1**A,B). Then, we conducted RNA sequencing (RNA‐seq) to explore other intrinsically relevant changes further. As shown in Figure 1C, there were 166 genes upregulated and 184 genes downregulated significantly in the low‐glc group as compared with the control group. Moreover, Figure 1D and Figure S1A (Supporting Information) showed that genes involved in the glycolysis pathway were significantly enriched in the low‐glc group under gene set enrichment analysis (GSEA), while genes involved in the NADP binding pathway were downregulated. In addition, it is worthwhile to note that genes in copper ion binding pathways were significantly downregulated in the low‐glc group, indicating an imbalance in copper metabolism (Figure 1D). Specifically, glycolysis‐related genes such as hexokinase2 (Hk2), enolase 2 (Eno2), copper ion transporter gene solute carrier family 31 member 2 (Slc31a2) and mitochondrial copper ion transporter gene solute carrier family 25 member 3 (Slc25a3) were upregulated, copper ion binding genes such as recombinant superoxide dismutase 3, extracellular (Sod3), amine oxidase [copper containing] 2 (Aoc2) were downregulated, which were further verified by our real time quantitative PCR (RT‐qPCR) experiments (Figure 1E,F; Figure S1B, Supporting Information). Our results suggested that low glucose could reinforce glycolysis and affect intracellular copper homeostasis in LLC cells.

The imbalance of copper metabolism caused by low glucose may be a potential enhancement for cell cuproptosis. Elesclomol (Es), a copper ionophore that could transport Cu into cellular mitochondria to induce LLC cell cuproptosis (Figure S1C–E, Supporting Information), was employed to evaluate the combining potential of cuproptosis and glucose starvation. Cell viability assays showed that low‐glc and Es‐Cu could limit LLC cell proliferation individually, while combined treatment of low‐glc and Es‐Cu had a better inhibition effect (Figure S1F, Supporting Information). Meanwhile, the intracellular reactive oxygen species (ROS) levels in LLC cells displayed by flow cytometry were increased when treated with Es‐Cu, while low‐glc + Es‐Cu induced more than alone (Figure 1G,H). In comparison with low‐glc treated group, Es‐Cu treatment could induce higher expression of PD‐L1 on LLC cells. More importantly, the combinational treatment of low‐glc and Es‐Cu elevated PD‐L1 expression in cells compared to those treated with low‐glc or Es‐Cu alone (Figure 1I,J). The above results indicated that combining glucose starvation and cuproptosis could be a promising combination therapy with αPD‐L1 immunotherapy against lung cancer.

### 2.2 Preparation and Characterization of Nanomedicine

**Figure 2:**
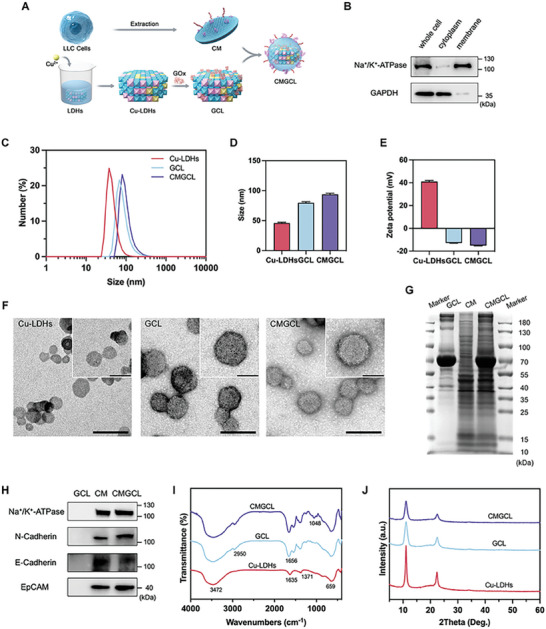
Preparation and characterization of CMGCL. A) Scheme illustration of GOx loading into Cu‐LDHs with CM camouflaging. B) Expression analysis of membrane‐specific protein Na^+^/K^+^‐ATPase and cytoplasm‐specific protein GAPDH in LLC whole cell, cytoplasm, and membrane by WB. C) Representative particle size distributions of Cu‐LDHs, GCL, and CMGCL measured by dynamic lighting scattering (DLS). D) Average size of Cu‐LDHs, GCL, and CMGCL measured by DLS. E) Average zeta potential of Cu‐LDHs, GCL and CMGCL. F) Representative TEM images of Cu‐LDHs, GCL, and CMGCL. Scale bar: 100 nm. Internal scale bar: 50 nm. G) Coomassie blue staining of SDS‐PAGE for GCL, CM, and CMGCL. H) WB analysis for membrane‐specific protein Na^+^/K^+^‐ATPase, N‐Cadherin, E‐Cadherin, and EpCAM in GCL, CM, and CMGCL. I) FT‐IR spectra of Cu‐LDHs, GCL, and CMGCL. J) XRD patterns of Cu‐LDHs, GCL, and CMGCL. Data are shown as mean ± SEM; *n* = 3.

To simultaneously achieve the combination effect of glucose starvation and cuproptosis, we designed a cancer cell‐targeted nanomedicine as shown in the illustration (**Figure** **2**A). The green synthesized Cu‐LDHs had LDHs‐typical X‐ray diffraction (XRD) patterns, indicating the successful Cu doping into the crystal layered framework (Figure S2A, Supporting Information). X‐ray photoelectron spectroscopy (XPS) verified the existence of Cu, Mg, Al, O elements in the Cu‐LDHs (Figure S2B, Supporting Information). More specifically, the fitting analysis peaks of Cu2p shown in Figure S2C (Supporting Information) proved the existence of mixed valence states, and that most Cu ions (75.22%) were divalent with characteristic Cu^2+^ satellite peaks at 942.47 and 961.84 eV. In addition, the specific copper content in Cu‐LDHs were determined by the inductively coupled plasma experiment as 5.8%.

Subsequently, cell membranes of LLC cells (CM) were extracted, and the successful isolation was verified by western blot (WB) analysis of cytoplasm‐specific protein GAPDH and membrane‐specific protein Na^+^/K^+^‐ATPase levels in the whole cell, CM and cytoplasm (Figure 2B). Meanwhile, the GOx was loaded onto Cu‐LDHs at an optimized mass ratio of GOx: Cu‐LDHs = 1:16 (Figure S3A,B, Supporting Information). Compared with Cu‐LDHs, the average particle size of GOx‐Cu‐LDHs (GCL) increased from 45.97 nm (polymer dispersity index (PDI): 0.225) to 79.77 nm (PDI: 0.151), while zeta potential decreased from 41.01 to −12.85 mV. After surface coating of CM (Figure S3C,D, Supporting Information), the CM‐GOx‐Cu‐LDHs (CMGCL) reached ≈93.93 nm (PDI: 0.197) and zeta potential of −15.07 mV (Figure 2C–E). According to the transmission electron microscopy (TEM) imaging, CMGCL displayed a typical hexagonal morphology as Cu‐LDHs and GCL, and a CM layer of ≈10 nm (Figure 2F), indicating successful surface coating with cell membranes.

As shown in Figure 2G, the protein profiles in CMGCL not only retained bands of GCL but also possessed similar protein bands to CM, further proving successful GOx loading and membrane proteins translocating in CMGCL. Besides, membrane‐specific protein Na^+^/K^+^‐ATPase, cancer cell membrane‐specific protein N‐Cadherin, E‐Cadherin, EpCAM were identified in CM and CMGCL (Figure 2H). The Fourier transform infrared (FT‐IR) spectra of each group further proved the loading of GOx and CM on Cu‐LDHs (Figure 2I). All samples exhibited characteristic broad band centrally located at 3472 cm^−1^, representing the O─H groups from LDHs layer and interlay H_2_O, and the strong absorption peak in 800–480 cm^−1^ was ascribed to the M─O vibrations and M─O─M bending (M = Cu, Mg, Al) of LDHs layer. Additionally, the obvious peaks at 1540, 1656, and 2950 cm^−1^ in GCL and CMGCL represented stretching vibrations of C═O, C─H, and N─H bonds, which were the characteristics of GOx and CM. The extra band centered at 1048 cm^−1^ observed in CMGCL presumably assigned to P─O─C bond of phospholipids in CM. Moreover, the identical typical peaks in XRD patterns suggested that the crystal framework of Cu‐LDHs was well preserved in GCL and CMGCL (Figure 2J). Taken together, these results verified the successful synthesis of the designed CMGCL.

### 2.3 Effective Catalytic Properties of CMGCL

**Figure 3:**
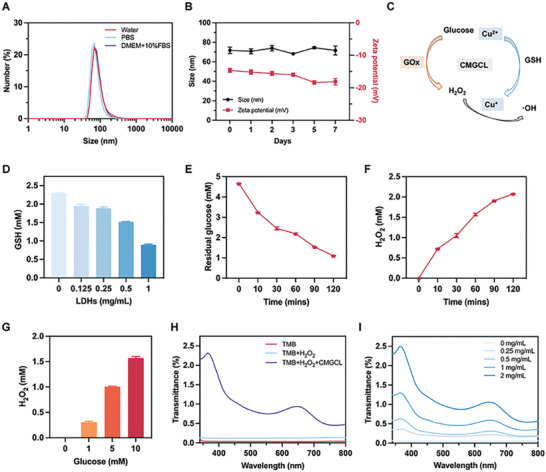
Effective catalytic properties of CMGCL. A) Particle size distributions of CMGCL kept in deionized water, PBS or DMEM + 10% FBS for 24 h, measured by DLS. B) Average size and zeta potential of CMGCL in PBS for 7 days. C) Schematic illustration of cascade catalytic reactions induced by CMGCL. D) GSH content after incubation with CMGCL at different concentrations. E) Residual glucose content after reaction with CMGCL at different time points. F) H_2_O_2_ generation from the reaction between CMGCL (GOx: 50 µg mL^−1^) and glucose (10 mm) at different time points. G) H_2_O_2_ generation from reactions between CMGCL (GOx: 50 µg mL^−1^) and different glucose concentrations for 1 h. H) UV−vis absorption spectra of TMB after co‐incubated with or without H_2_O_2_ or CMGCL. I) Oxidation of colorless TMB by •OH generated from reaction between H_2_O_2_ and different concentrations of CMGCL. The blue‐colored oxTMB had representative absorbance peaks peaked at 370 and 652 nm. Data are depicted as mean ± SEM; *n* = 3.

For further study, we first examined the stability of GCL and CMGCL. As shown in **Figures** **3**A and S4A (Supporting Information), both GCL and CMGCL exhibited similar particle size distributions in deionized water, PBS and DMEM + 10% FBS. In addition, the particle size and zeta potential remained relatively stable in PBS for 7 days (Figure 3B; Figure S4B, Supporting Information). To explore the pH‐responsive copper ions release ability, the CMGCL was incubated in PBS solution with different pH values, the results showed that the copper release rate was much faster under pH 5.6 condition within 24 h than pH 7.4 condition (Figure S4C, Supporting Information). Figure 3C illustrates the cascade catalytic reactions of CMGCL, including the GSH consumption caused by redox of copper ions, GOx‐catalysis with glucose and enhanced •OH generation via Fenton‐like reactions. The GSH content decreased gradually after incubation with different CMGCL concentrations, which could be owing to the redox reaction of Cu^+^/Cu^2+^ electron pair (Figure 3D).

Meanwhile, the glucose‐responsive properties of GOx in CMGCL were conducted by incubating glucose with CMGCL. Notably, the residual glucose content sharply decreased while the generated H_2_O_2_ quickly increased within 120 min, indicating the high catalytic efficiency (Figure 3E,F). Additionally, the glucose oxidation reaction showed a concentration‐dependent manner, with more H_2_O_2_ generated after a 1‐h reaction with higher glucose level (Figure 3G). More importantly, CMGCL was able to achieve a Fenton‐like response by catalyzing H_2_O_2_ into •OH in a concentration‐dependent manner as determined by a 3,3′,5,5′‐tetramethylbenzidine (TMB) colorimetric assay (Figure 3H,I). These reactions induced by CMGCL indicated the feasibility of subsequent applications for killing lung cancer cells.

### 2.4 In Vitro Cytotoxicity of CMGCL for Lung Cancer Cells

**Figure 4:**
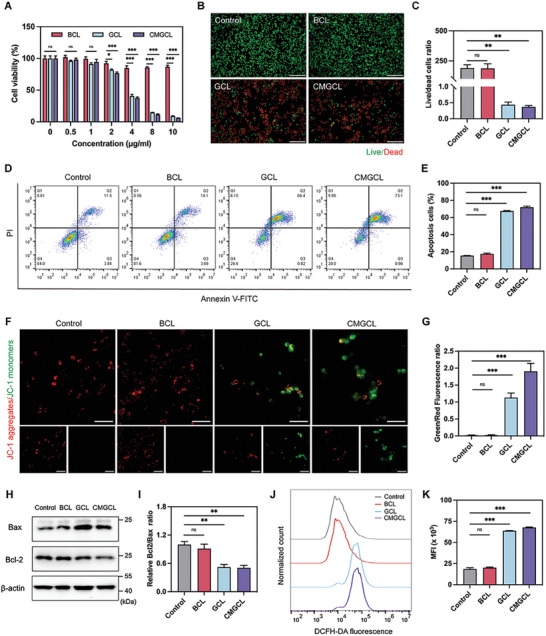
In‐vitro cytotoxicity of CMGCL for lung cancer cells. A) Cell survival rate of LLC cells after treated with BCL, GCL, and CMGCL at different concentrations for 12 h. B) Representative fluorescence images of LLC cells co‐stained with Calcein AM (green, live cells) and Propidium Iodide (PI) (red, dead cells) after treatments with BCL, GCL, and CMGCL for 12 h. Scale bar: 100 µm. C) The ratio of live cells to dead cells in each group was quantitatively analyzed. D,E) The population of apoptosis cells (Q2+Q3) via Annexin V‐FITC/PI double‐staining assay using flow cytometry analysis. F,G) Representative fluorescence images and quantitative analysis of JC‐1 aggregates (red) and JC‐1 monomers (green) in LLC cells after different treatments for 4 h. Scale bar: 50 µm. H) WB analysis for Bcl‐2 and Bax protein expressions in LLC cells after different treatments. I) Relative Bcl‐2/Bax protein expression ratio of each group. The expression levels of the proteins were normalized to that of β‐actin. J,K) Representative flow cytometry histograms and quantitative analysis of intracellular ROS levels of LLC cells after different treatments. All data are depicted as mean ± SEM; *n* = 3. ns, not significant, ^*^ *p* < 0.05,^**^ *p* < 0.01, ^***^ *p* < 0.001.

Then, the anti‐tumor activities of these nanoparticles were evaluated on LLC cells in vitro. As shown in **Figure** **4**A, BCL had a mild effect on LLC cell growth (with nearly 90% survival cells at 10 µg mL^−1^ treatment), while GCL and CMGCL exhibited potent cytotoxicity on LLC cells, showing less than 50% surviving cells over 4 µg mL^−1^ treatment. These findings indicated the synergistic inhibition mediated by the starvation effect and Fenton‐like reaction. Similarly, GCL and CMGCL treatments also showed the most significant number of dead cells (red), while almost all cells were alive after the BCL treatment (green) via live‐dead cell staining (Figure 4B,C). In addition, the percentage of apoptosis cells generated by BCL, GCL, and CMGCL was examined via flow cytometry analysis, and were 17.79%, 67.22%, and 74.06%, respectively (Figure 4D,E).

To explore the potential mechanisms involved, the mitochondrial membrane potential (MMP) of LLC cells after different treatments was first measured to evaluate the permeabilization of mitochondrial outer membrane, which acts as a central role in cell death and early apoptosis.^[^ ^23^ ^]^ Clearly, the control and BCL groups displayed high MMP with red solid fluorescence (JC‐1 aggregates), while the GCL and CMGCL groups changed a lot into green fluorescence (JC‐1 monomers), representing much lower MMP and mitochondria dysfunction (Figure 4F,G). In addition, compared to the control and BCL groups, GCL and CMGCL treatments induced increased levels of Bax (pro‐apoptotic protein) and suppressed expression of Bcl‐2 (anti‐apoptotic protein), indicating the increase of mitochondrial membrane permeability and evident cell apoptosis (Figure 4H,I). Notably, cell mitochondria dysfunction is always related to elevated intracellular ROS levels. The results showed that GCL and CMGCL treatments significantly increased intracellular ROS levels, while incubation of BCL had a negligible effect (Figure 4J,K). These findings well demonstrated the cytotoxicity of CMGCL on lung cancer cells by mitochondria‐related cell death.

### 2.5 Cuproptosis Activation and PD‐L1 Upregulation Mediated by CMGCL

**Figure 5:**
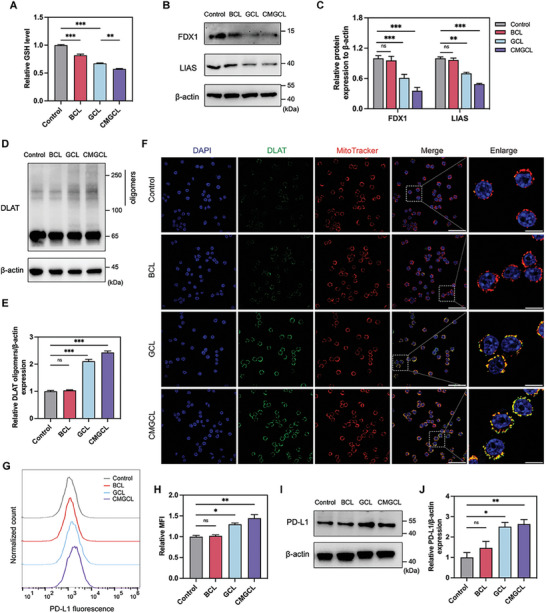
Cuproptosis activation and PD‐L1 upregulation mediated by CMGCL. A) Relative GSH level in LLC cells after treated with BCL, GCL, and CMGCL. B) WB analysis of FDX1 and LIAS protein expression after different treatments in LLC cells. C) Quantitative analysis of protein expressions after normalized by the levels of β‐actin. D) WB for DLAT and its oligomers of LLC cells with indicated treatments. E) Quantitative analysis of DLAT oligomers in different groups after normalized by the levels of β‐actin. F) Representative CLSM images of DLAT (green) expression in LLC cells with various treatments. Cell mitochondria were co‐stained with MitoTraker (red) and nuclei were co‐stained with DAPI (blue). Scale bar: 50 µm; Enlarged scale bar: 10 µm. G,H) Representative flow cytometry histograms and quantitative analysis of PD‐L1 expression on LLC cells after different treatments. I) PD‐L1 expression level changes in LLC cells by WB analysis. J) Quantitative analysis of PD‐L1 in different groups after normalized by the levels of β‐actin. Data are presented in mean ± SEM; *n* = 3. ns, not significant, ^*^ *p* < 0.05, ^**^ *p* < 0.01, ^***^ *p* < 0.001.

By providing excess copper ions, GSH‐depleting capability and mitochondria‐related cell death, CMGCL may contribute to copper‐dependent cell death, cuproptosis.^[^ ^24^ ^]^ As shown in **Figure** **5**A, additions of BCL, GCL, and CMGCL could all consume the GSH content inside LLC cells significantly, indicating the redox of Cu ions with GSH, thus effectively weakening the antioxidant capacity of lung cancer cells and potentially triggering following cuproptosis. Notably, the relative GSH level in GCL and CMGCL groups decreased more than BCL group, which might imply the amplifying effect. To verify the hypothesis, the changes of key proteins in cuproptosis pathway, including ferredoxin 1 (FDX1), lipoic acid synthetase (LIAS) and dihydrolipoyl transacetylase (DLAT), were determined. After GCL and CMGCL treatment, FDX1 and LIAS in LLC cells were downregulated significantly (Figure 5B,C), while DLAT oligomers were strongly enhanced (Figure 5D,E). Moreover, as showed by confocal laser scanning microscopy (CLSM) images, LLC cells treated with GCL or CMGCL exhibited apparent DLAT foci co‐located with mitochondria labeled with MitoTracker (Figure 5F; Figure S4D, Supporting Information). These results indicated that CMGCL could induce LLC cell cuproptosis obviously.

Since glucose starvation and cuproptosis had synergistic effect on PD‐L1 upregulation on LLC cells (Figure 1I,J), we further studied the potential effects of different nanomedicine on PD‐L1 expression at the cellular level. The flow cytometry analysis confirmed BCL had negligible effect on PD‐L1 level, while GCL and CMGCL could obviously induce PD‐L1 overexpression on LLC cells (Figure 5G,H). In addition, WB analysis also showed that GOx‐containing Cu‐LDHs (GCL and CMGCL) significantly increased PD‐L1 expression (Figure 5I,J), offering an opportunity for a αPD‐L1 combined immunotherapy.

### 2.6 Precise Tumor‐Targeted Efficacy of CMBCL via CM Camouflage

**Figure 6:**
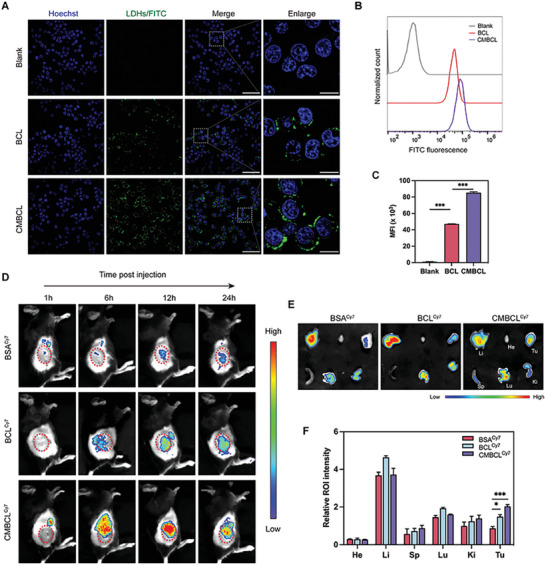
Precise tumor‐targeted efficacy of CMBCL via CM camouflage. A) Representative CLSM photographs of LLC cells after incubated with FITC‐labeled BCL or CMBCL (green) for 4 h at 37 °C. Cell nuclei were co‐stained with Hoechst 33342 (blue). Scale bar: 50 µm. Enlarged scale bar: 10 µm. B,C) Representative flow cytometry histograms and semi‐quantitative analysis of intracellular uptake of FITC‐labeled BCL or CMBCL by LLC cells after 4 h incubation at 37 °C. D) In vivo fluorescence images of LLC tumor‐bearing mice after intravenously injected with BSA^Cy7^, BCL^Cy7^, and CMBCL^Cy7^ at different time intervals. E,F) Ex vivo fluorescence pictures and relative quantitative analysis of dissected major organs and tumors post 24 h administration. He: Heart. Li: Liver. Sp: Spleen. Lu: Lung. Ki: Kidney. ROI: region of interest. Data are presented in mean ± SEM; *n* = 3. ^*^ *p* < 0.05, ^***^ *p* < 0.001.

Cancer cell membrane‐camouflaged nanomaterials possess the potential to achieve homotypic targeting via membrane‐specific affinity to parental cells.^[^ ^19a^ ^]^ As observed by CLSM images, more FITC‐labeled CMBCL were observed in cell cytoplasm than FITC‐labeled BCL, indicating the promoted cell‐targeted efficiency via CMBCL (**Figure** **6**A). Furthermore, flow cytometry analysis also showed the same results, with ≈1.8‐fold fluorescence intensity increasing after CM coating (Figure 6B,C). Such enhanced cellular internalization may be contributed by the homologous and rapid fusion of CM with LLC cancer cells.

To explore the tumor targeting ability and biological distribution in vivo, LLC tumor‐bearing mice were photographed after intravenous administrations of BSA^Cy7^, BCL^Cy7^, or CMBCL^Cy7^ at different time points. Compared to free BSA^Cy7^, Cy7‐labled nanoparticles could effectively accumulate at tumor sites with higher fluorescence. Especially, CMBCL^Cy7^ group was the highest for the whole time, reaching a peak at 12 h and lasting until 24 h (Figure 6D). As shown in the ex vivo images, the nanoparticles mainly accumulated in liver, tumor, lung, and kidney at 24 h post‐administration. The relative tumor fluorescence intensities of BCL^Cy7^ and CMBCL^Cy7^ groups were 1.7‐fold and 2.3‐fold that of BSA^Cy7^ group, respectively (Figure 6E,F). Taken together, the improved accumulation at tumor sites exhibited the pronounced tumor homing ability of CM‐coated nanoparticles.

### 2.7 Effective Anti‐Tumor Effects Against LLC Tumors of CMGCL In Vivo

**Figure 7:**
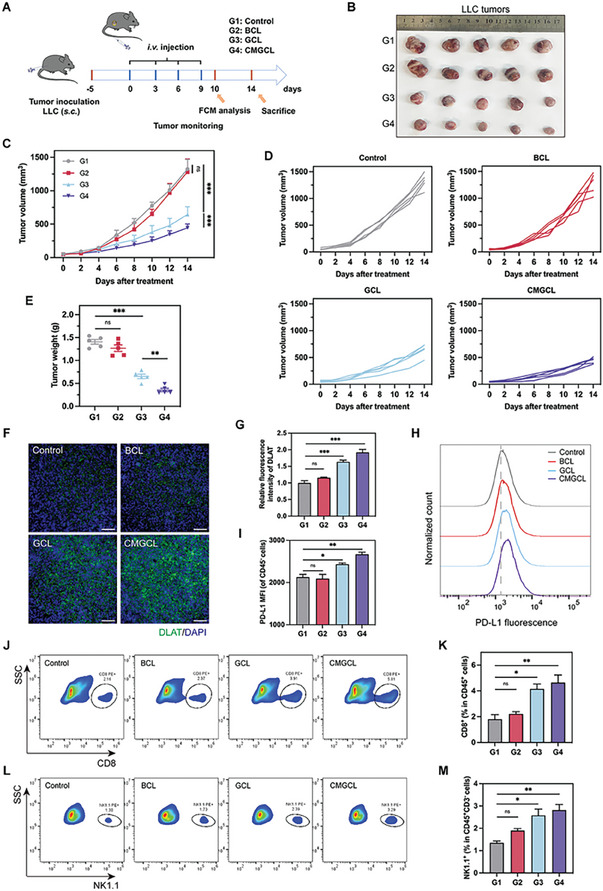
Effective anti‐tumor effects against LLC tumors of CMGCL. A) Schematic diagram of the experimental schedule for treatment of the subcutaneous LLC tumor model. FCM, flow cytometry. B) Photographs of dissected tumors on day 14 following different treatment. C) Tumor volume curves of subcutaneous LLC tumors during the experiments (*n* = 5). D) Individual tumor volume curves of different groups during the treatments. E) Average tumor weight in different groups on day 14 (*n* = 5). F) Representative fluorescence images DLAT (green) expression in different tumor sections. Cell nuclei were co‐stained with DAPI (blue). Scale bar: 50 µm. G) Relative fluorescence intensity of DLAT (green) expression in different groups (*n* = 3). H) Representative flow cytometry histograms of PD‐L1 expression levels in CD45^−^ cells of LLC tumor lysates. I) Quantitative analysis of mean PD‐L1 fluorescence intensities in CD45^−^ cells for different groups (*n* = 3). J) Representative flow cytometry profiles for the population of CD8^+^CD45^+^ cells in LLC tumors with different treatments. K) Quantitation for the percentages of CD8^+^CD45^+^ cells in each group (*n* = 3). L) Representative flow cytometry profiles for the population of NK1.1^+^CD3^−^CD45^+^ cells in LLC tumors with different treatments. M) Quantitation for the populations of NK1.1^+^CD3^−^CD45^+^ cells in tumors of each group (*n* = 3). G1, Control. G2, BCL. G3, GCL. G4, CMGCL. Data are depicted as mean ± SEM. ns, not significant, ^*^ *p* < 0.05, ^**^ *p* < 0.01, ^***^ *p* < 0.001.

Then, the in vivo anti‐tumor efficacy of CMGCL was evaluated in LLC tumor‐bearing mice (**Figure** **7**A). According to the tumor images and tumor growth curves, BCL treatment had negligible inhibition effect with the same expanding growth tendency as the control group. In contrast, GCL treatment showed a significant anti‐tumor effect (51.3% tumor inhibition rate) compared to PBS treatment, while CMGCL exhibited stronger suppression with an inhibition rate of 66.4% (Figure 7B–D). Tumor weights also showed the same trend in terms of tumor suppression (Figure 7E). Subsequently, histological evaluation of tumor sections using the hematoxylin and eosin (H&E), Ki‐67 immunochemistry staining and TdT‐mediated dUTP nick‐end labeling (TUNEL) assay were applied to further analyze therapeutic effects. The results verified the strongest anti‐proliferation and pro‐apoptotic capabilities of the CMGCL group (Figure S5A–C, Supporting Information). Thus, these results revealed the superior anti‐tumor effect by CMGCL. In the meantime, the efficiencies of different treatments in generating cuproptosis were detected as well. The CMGCL treatments increased DLAT signals, which clearly demonstrated the activation of curproptosis in vivo (Figure 7F,G).

As the tumor immune micro‐environment (TIME) is of vital importance for tumor therapy, the status of TIME, such as PD‐L1 level and tumor‐infiltrating immune cells proportions, was further assessed. The histogram graphs illustrated that the PD‐L1 expression in CD45^−^ cell populations greatly enhanced after CMGCL treatment (Figure 7H,I; Figure S6A, Supporting Information). Moreover, the frequency of tumor‐infiltrating CD8^+^ T cells (CD45^+^CD8^+^) and NK cells (CD45^+^CD3^−^NK1.1^+^) upregulated significantly after CMGCL treatment, which was 2.57‐fold and 2.08‐fold than the control group (Figure 7J–M; Figure S6B,C, Supporting Information), indicating that CMGCL could activate anti‐tumor immune response partially. In summary, CMGCL could mediate potent anti‐cancer effects via multiple strategies ranging from starvation to curproptosis, and also provide a possibility for sensitizing αPD‐L1 therapy by increasing PD‐L1 expression in vivo.

Additionally, the biosafety of these treatments was evaluated on day 14. As displayed by the results of H&E images, no significant morphological changes were observed in the major organs from groups after different therapies, compared to the control group (Figure S7A, Supporting Information). Moreover, the blood physiological and biochemical indicators of each group were detected. The results showed that the aspartate aminotransferase (AST), alanine transaminase (ALT), blood urea nitrogen (BUN) and creatinine (CR), lactate dehydrogenase (LDH) and creatine kinase (CK) in nanoparticle treated mice had no significant difference from those treated with PBS (Figure S7B–D, Supporting Information). The body weight of mice with different treatments changed during the time, while the difference between groups had no statistical significance (Figure S8A, Supporting Information). These results all suggested no evident systemic side effects of these nanoparticles.

### 2.8 Sensitization of αPD‐L1 Immunotherapy via CMGCL Combined Treatment

**Figure 8:**
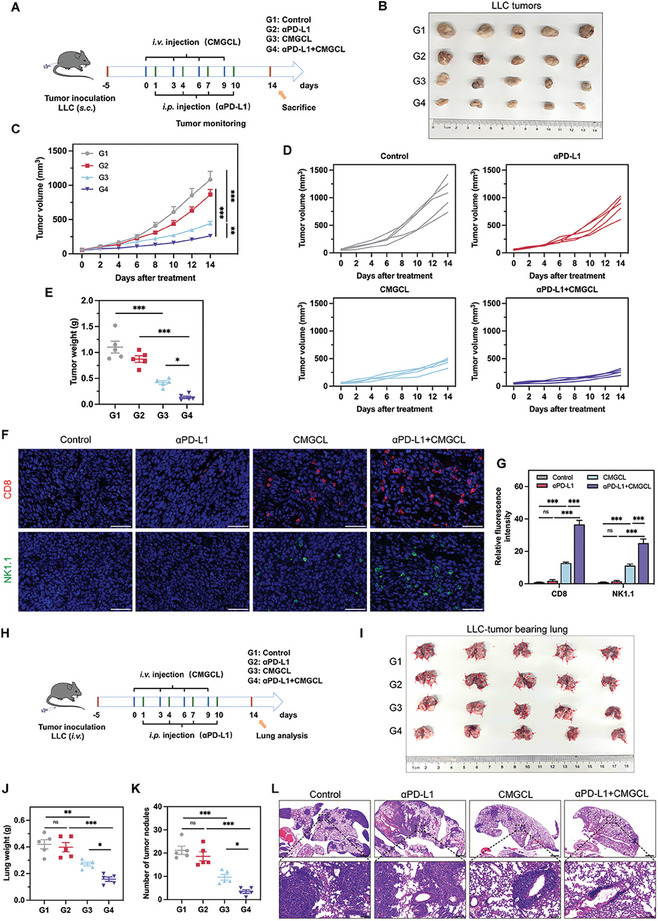
Sensitization of αPD‐L1 immunotherapy via CMGCL combined treatment. A) Schematic illustration of the experimental schedule for treatment of the subcutaneous LLC tumor model. B) Visualization of tumor specimens on day 14 following different treatments. C) Tumor volume curves of LLC tumor‐bearing mice in each group (*n* = 5). D) Individual tumor volume curves. E) Average tumor weight in different groups on day 14 (*n* = 5). F,G) Representative fluorescence images and relative fluorescence intensity of CD8 (red) and NK1.1 (green) in tumor sections of different groups (*n* = 3). Cell nuclei were co‐stained with DAPI (blue). Scale bar: 50 µm. H) Schematic illustration of the experimental schedule for LLC tumor lung metastatic model treatment. I) Digital photos of lungs in each group after 14 days of treatments. J) Lung weight of each group at day 14 (*n* = 5). K) Numbers of lung metastatic tumor nodules (*n* = 5). L) Representative H&E staining images of lungs in different groups. Scale bar: 1000 µm. Enlarged scale bar: 100 µm. G1, Control. G2, αPD‐L1. G3, CMGCL. G4, αPD‐L1 + CMGCL. Data are depicted as mean ± SEM. ns, not significant, ^*^ *p* < 0.05, ^**^ *p* < 0.01, ^***^ *p* < 0.001.

Given CMGCL's capability in upregulating PD‐L1 expression on tumor cells, CMGCL could be potentially applied as a sensitizer for αPD‐L1 immunotherapy, and generate synergistic effects via a combined therapy. Therefore, subcutaneous LLC tumor‐bearing mice models were treated following the procedure portrayed in **Figure** **8**A. The body weight among groups exhibited no any significant difference during the monitoring period, indicating no systemic toxicity at these doses (Figure S8B, Supporting Information). As displayed by the dissected tumor photographs and tumor growth curves, monotherapy of αPD‐L1 exhibited limited inhibition against lung cancer, while CMGCL + αPD‐L1 markedly suppressed LLC tumor growth with the best anti‐tumor efficacy (Figure 8B–D). In consistency, the tumor weight of each group showed the same tendency as above (Figure 8E). Furthermore, the most intensive CD8^+^ T cells and NK1.1^+^ cells infiltrated tumor tissues from mice treated with CMGCL + αPD‐L1 among all the groups (Figure 8F,G). To further prove the combination therapeutic effect of CMGCL and αPD‐L1 on lung cancer, a lung metastatic mice model was established by intravenously injected with LLC cells and divided into four groups with different treatments (Figure 8H). On 14 days post‐first administration, lung metastatic foci were evidently seen in the control and αPD‐L1 groups. In contrast, CMGCL group showed a significant improvement, while CMGCL + αPD‐L1 group had much less and smaller metastasis foci with the lightest lung weight (37.6% weight of the control group and 39.7% weight of the αPD‐L1 group) (Figure 8I,J). The H&E images of lung sections exhibited the same results, and the lung metastatic nodules were minimal (average of 3.4 nodules) in the combination group (Figure 8K,L). The above findings declared the feasibility of combining CMGCL and αPD‐L1 for lung cancer therapy.

## 3 Discussion

αPD‐L1 therapy is one of the most hopeful immunotherapies for lung cancer, and tumor PD‐L1 expression is an essential determinant.^[^ ^4b^ ^]^ However, the immune response is dynamic, and there is still a large part of lung cancer patients who are poor responders, especially patients carrying low‐expressed PD‐L1. Moreover, those receiving αPD‐L1 monotherapy would generally develop resistance or irAEs to varying degrees, eventually restraining the therapeutic effect.^[^ ^3^, ^4^ ^]^ Therefore, a novel, highly effective, and biocompatible combination therapy would be a viable method to enhance the efficacy of αPD‐L1 treatment for lung cancer.^[^ ^25^ ^]^

In this study, we noticed that low glucose could significantly upregulate PD‐L1 levels on LLC lung cancer cells and activate the glycolysis pathway (Figure 1A–D), similar to previous findings.^[^ ^26^ ^]^ Meanwhile, according to our RNA‐seq findings, low glucose treatment may influence intracellular copper balance (Figure 1D–F), inspiring us to further intervene intracellular copper content. Studies have shown that limiting glucose could induce cell oxidative stress by decreasing NADPH production.^[^ ^27^ ^]^ Accumulated intracellular copper can also cause oxidative stress and lead to cuproptosis, which has been systemically verified as a potent cancer therapy.^[^ ^14b^ ^]^ By combining low glucose treatment and cuproptosis, the intracellular ROS generation and PD‐L1 levels in LLC cells could be significantly enhanced simultaneously (Figure 1G–J), which would synergistically sensitize αPD‐L1 immunotherapy. To achieve that, an intelligent delivering platform with precise targeting would greatly promise the efficiency of combined therapy.

Nanomedicines have shown remarkable potential as powerful delivery agents for treating various cancers due to their desirable physicochemical features.^[^ ^28^ ^]^ With attractive biocompatibility and tumor‐targeted efficiency, cancer cell membrane‐coated nanomedicine has gradually become a promising strategy for cancer synergistic therapies.^[^ ^29^ ^]^ Here, we designed and successfully synthesized an intelligent delivery system CMGCL by camouflaging LLC cell membranes onto GOx loading Cu‐LDHs (Figure 2A). The CMGCL could induce cascade catalytic reactions to achieve glucose starvation and enhanced Fenton‐like reaction (Figure 3C), as the basis of subsequent cellular and animal experiments. More specifically, our results verified that CMGCL‐induced LLC cell death was mainly due to mitochondria dysfunction and cuproptosis (Figure 4F–K and Figure 5B–F). Generally, the Cu^+^ accumulated inside mitochondria can cause mitochondria damage and cuproptosis via the lipoylated mitochondrial enzymes aggregation and Fe‐S cluster protein loss.^[^ ^13^, ^24^ ^]^ After internalization, excess intracellular ROS and Cu^+^ were generated from CMGCL via cascade catalytic reactions, and accumulated in mitochondria, strongly inhibiting cancer cells. Notably, BCL treatment showed no significant changes in LLC cells at the same concentration, demonstrating the importance of every step in the cascade catalytic reactions. Compared to those Cu‐based nanomaterials alone (e.g., copper oxide nanoparticles), this cascade amplifying effect enabled less Cu dosage, thus avoiding the potential side effects of excessive metal ions.^[^ ^30^ ^]^

In addition, CM‐coated nanomedicine has homologous targeting capability for cancer sites, with superior biocompatibility.^[^ ^19a^ ^]^ Our findings indicated that CMGCL retained the intact membrane protein profiles characteristic of LLC cells, underscoring its potential for therapeutic applications (Figure 2G,H). The cellular uptake and in vivo imaging experiments verified that CM‐coating well improved the tumor targeting efficiency of Cu‐LDHs both in virto and in vivo (Figure 6). Compared to those bare nanomaterials or nanoparticles modified with targeting proteins (e.g., folate receptor 1, transferrin), CM camouflage would exhibit better targeting to tumor sites in lung cancer mouse models with its integrity of membrane proteins.^[^ ^31^ ^]^ Moreover, the tumor‐targeted capacity of CMGCL resulted in enhanced targeting efficiency and tumor inhibition effect in LLC mouse models compared to uncoated GCL. Specifically, CMGCL improved ≈15% tumor inhibition rate than GCL (Figure 7C–E), with more cuproptosis activation and PD‐L1 upregulation at tumor sites (Figure 7F–H), indicating the necessity of CM coating. Furthermore, CMGCL showed great biosafety in our mice models (Figure S7, Supporting Information). The unique characteristics of “mimicking nature” enabled CM‐NPs superiority to other nanomaterials.^[^ ^19a^ ^]^ The great biocompatibility and pH‐responsive features also make LDHs an excellent core of CM‐NPs.^[^ ^20a^ ^]^

Sensitization of lung cancer αPD‐L1 therapy is of great vital for improving lung cancer immunotherapy. Our results proved that glucose starvation could upregulate PD‐L1 expression on LLC cells synergistically with cuproptosis. Nanoparticles inducing cell cuproptosis have shown a combinational anti‐tumor effect with αPD‐L1 therapy in bladder cancer and colorectal cancer.^[^ ^32^ ^]^ However, to our best knowledge, there is no such study to combine glucose starvation and cuproptosis with αPD‐L1 therapy for lung cancer. Based on our results, the combinational strategy provided more promising therapeutic effects than monotherapy. More specifically, CMGCL accomplished glucose starvation and enhanced cell cuproptosis effect in cancer cells, then significantly promoted PD‐L1 upregulation both in virto and in vivo. Moreover, the therapy mediated by CMGCL triggered more CD8 T cells and NK cells infiltration into tumors, further enhancing the possibility of immunotherapy sensitization (Figure 7J–M). Previous research found that the tumor‐associated antigens on cancer cell membranes might also elicit anti‐tumor immune response.^[^ ^19b^ ^]^ With the attendance of LDHs, desirable nano‐adjuvant, it can be believed that certain immunity against tumors has been evoked systemically, either from tumor‐draining lymph nodes or the peripheral immune system. The upregulation of PD‐L1 expression on the tumor epitope and the increased number of immune cells ultimately achieved immune enhancement. Thus, significant therapeutic effects were generated by CMGCL combined with αPD‐L1 therapy in both subcutaneous and lung metastatic models (Figure 8). Traditional combination therapies for αPD‐L1 therapy in clinical practice are generally accompanied by inevitable side effects. Compared to the direct damages caused by chemotherapeutic drugs or radiotherapy, the applications of smart medicine, such as bio‐responsive nanomedicine, would debilitate the ramifications to a large extent. The sensitization of αPD‐L1 therapy by CMGCL exhibited great potential since its ease of use and cascade amplifying effects than alone, which represented less dosage with better efficiency, thus avoiding those possible side effects.

## 4 Conclusion

In summary, this study shows a new aspect of the connection between glucose metabolism and intracellular copper homeostasis. It enhances understanding of glucose starvation and cuproptosis therapies in promoting lung cancer immunotherapy, especially αPD‐L1 therapy. More importantly, a novel biomimetic nanomedicine CMGCL was successfully synthesized to combine cancer starvation/cuproptosis/immunotherapy in lung cancer, demonstrating great potential as a promising strategy for αPD‐L1 immunotherapy.

## Conflict of Interest

The author declare no conflict of interest.

## Supporting information



Supporting Information

## Data Availability

The data that support the findings of this study are available from the corresponding author upon reasonable request.
