# Supplementary material for: A Smart Nanomedicine Unleashes a Dual Assault of Glucose Starvation and Cuproptosis to Supercharge αPD‐L1 Therapy
Source: Adv Sci (Weinh). 2024 Dec 4;12(4):2411378. doi: 10.1002/advs.202411378 (PMC11775525; doi:10.1002/advs.202411378)
Supplement: Supplementary file 1 — Supporting Information [file ADVS-12-2411378-s001.docx]

Supporting Information

A Smart Nanomedicine Unleashes a Dual Assault of Glucose Starvation and Cuproptosis to Supercharge αPD-L1 Therapy

Yiming Xu, Yuan Wu, Xinjie Zheng, Dongxue Wang, Hangqi Ni, Weiyu Chen*, Kai Wang*

Experimental section

1. **Materials and reagents**

DMEM, PBS, penicillin and streptomycin were obtained from KeyGEN Biotech (Jiangsu, China). Es was obtained from Selleck (Shanghai, China). MgCl_2_·6H_2_O, AlCl_3_·6H_2_O, NaOH and CuCl_2_·2H_2_O were purchased from Adamas Beta (Shanghai, China). GOx was obtained from Sigma-Aldrich. BSA, Coomassie Blue Fast Staining Solution and DCFH-DA were obtained from Beyotime (Shanghai, China). Sucrose, D-Glucose, GSH, TMB were acquired from Sangon Biotech (Shanghai, China). FITC was acquired from Aladdin (Shanghai, China). BSA-Cy7 was acquired from Ruixi Biotech (Xi’an, China). Hoechst 33342 was acquired from Invitrogen (USA). DAPI Fluoromount-G and MitoTracker Deep Red FM were acquired from Yeasen Biotech (Shanghai, China). CCK-8 solutions and chemiluminescence substrate were purchased from FDbio (Hangzhou, China). HiScript III All-in-one RT SuperMix and SYBR qPCR Master Mix were obtained from Vazyme Biotech (Nanjing, China). *InVivo*MAb anti-mouse PD-L1 (B7-H1) was purchased from BioXCell (USA). All primers were synthesized by Tsingke (Beijing, China). Antibodies for WB, immunofluorescence and immunohistochemistry were listed in Table S1. Flow cytometry antibodies were all purchased from BioLegend (USA).

1. **Cell lines and Animals**

LLC cells were originally obtained from American Type Culture Collection (ATCC). Cells were all cultured in complete DMEM containing 10% FBS, 100 units/mL penicillin and 100 μg/mL streptomycin and maintained at 37 °C with 5% CO_2_ in an incubator. Female C57BL/6J mice (5-8 weeks of age) were acquired from Gempharmatech Co. (Nanjing, China) and raised under specific pathogen-free conditions. The animal experiments were approved by The Institutional Animal Care and Use Committee of Zhejiang University.

1. **RNA-seq and bioinformatic analysis**

Total RNA of LLC cells was extracted using TRIzol reagent instructions. RNA-seq was performed by ANNOROAD Gene Technology (Beijing, China). In brief, the quality and quantity of RNA samples were detected by NanoPhotometer (IMPLEN, CA, USA) and Agilent 2100 RNA Nano 6000 assay kit (Agilent Technologies, CA, USA). A total amount of 1-3 μg RNA per sample was used as input material for the RNA sample preparations. Sequencing libraries were generated using VAHTS Universal V6 RNA-seq Library Prep Kit for Illumina (NR604-01/02) following the manufacturer’s recommendations and index codes were added to attribute sequences to each sample. The cluster generation and sequencing were performed on NovaSeq 6000 S4 platform with NovaSeq 6000 S4 Reagent kit V1.5 according to the vendor’s protocol.

The raw sequence data reported in this paper have been deposited in the Genome Sequence Archive in National Genomics Data Center,^[1]^ China National Center for Bioinformation / Beijing Institute of Genomics, Chinese Academy of Sciences (GSA: CRA018811) that are publicly accessible at https://ngdc.cncb.ac.cn/gsa.

Further bioinformatic analysis was conducted with the Solargenomics tools (https://www.solargenomics.com/) and the OmicStudio tools (https://www.omicstudio.cn/tool). GSEA analysis was conducted to identify the significant gene sets and pathways using the normalized gene expression using the GSEA v4.3.2 tool (www.broad.mit.edu/gsea/).

1. **RNA isolation and real time quantitative PCR (RT-qPCR)**

Total RNA was isolated from lysed LLC cells using an RNA-quick purification kit (ESScience, Shanghai, China) with the vendor’s protocol. The purity and concentration of RNA were detected by a Nanodrop ONE (Thermofisher, USA). Reverse transcription of RNA to cDNA was conducted and then RT-qPCR was performed on LightCycler 96 (Roche, Switzerland). β-actin was used as an internal control to quantify the relative expression of target genes. Sequences of all primers were listed in Table S2.

1. **Green synthesis of Cu-LDHs**

First, MgCl_2_ (0.6 M) and AlCl_3_ (0.2 M) were dissolved in deionized water and rapidly dropped into NaOH solution (0.4 M) under stirring. After 20 min, the formed Mg_3_Al-LDHs were harvested with centrifugation, washed twice by deionized water, and re-suspended in deionized water. The precursor Mg_3_Al-LDHs were sonicated and aged at room temperature. Then, a dropwise CuCl_2_ (40 mM) solution was added into the transparent Mg_3_Al-LDHs suspension under vigorous stirring for 4-8 hours to achieve Cu-substitution. Finally, Cu-LDHs were obtained through high-speed centrifugation (15000 rpm) and washed twice with deionized water. After gentle sonification, the transparent Cu-LDHs suspension was kept at room temperature for further use.

1. **Cell membranes isolation**

Cell membranes were isolated according to previously reported methods.^[2]^ In brief, LLC cells were collected, rinsed twice by PBS and suspended in an ice-cold TM buffer (10 mM Tris-HCl and 1 mM MgCl_2_, pH 7.4) solution with a density of 1 x 10^7^ cells/mL. Then, cells were disrupted by extruding through a mini-extruder for at least 35 times. Cell suspension with a final concentration of 0.25 M sucrose was centrifuged at 2000 g for 10 min in 4 °C. The supernatant was further centrifuged at 3000 g for 30 min in 4 °C. The cell membranes were obtained and purified by washing with ice-cold TM buffer containing sucrose (0.25 M) twice. The total protein content in purified membranes was determined by the bicinchoninic acid (BCA) protein quantification kit (Yeasen, Shanghai, China).

1. **Synthesis of GCL and CMGCL**

Equal volume of Cu-LDHs (2 mg/mL) solution was slowly dropped into GOx-BSA mixed solution (5 mg/mL) via vigorous stirring. After stirring for 30 min, the GCL were collected with centrifugation and resuspended in deionized water or PBS for further use.

To obtain membrane-coated nanoparticles, GCL solution was added dropwise into the cell membranes suspension with equal volume under vigorous stirring and then sonicated in an ice-water bath sonicator. Different mass ratios of cell membranes to LDHs (membranes: LDHs =1:1, 1:5, 1:10, 1:15, 1:20) were applied for optimization and ratio of 1:10 was used for subsequent experiments.

BCL and CMBCL were synthesized as the same.

1. **Characterization of nanoparticles**

The hydrodynamic particle size and zeta potential of different samples were detected by Zetasizer Advance (Malvern, UK). The morphology characteristics were visualized on a TEM (JEM1200EX, JEOL, Japan). The main elements in Cu-LDHs and the valence of doped copper were analyzed by XPS testing using Thermo escalab 250XI (Thermo Fisher Scientific, USA). The XRD patterns and FT-IR spectra of different nanoparticles were determined by D8 ADVANCE X-ray diffractometer (Bruker, Germany) and INVENIO S spectrometer (Bruker, Germany), respectively.

1. **Protein characterization of cell membranes and nanoparticles**

WB was used to verify the successful isolation of cell membranes. Lysates of LLC cells, cell membranes and cytoplasm extracted from LLC cells were separated using 10% or 12.5% SDS-PAGE and then transferred onto polyvinylidene fluoride membranes. Followed by being blocked, the membranes were incubated with the indicated primary antibody at 4 °C overnight. After incubation with the correspondent secondary antibody for 2 hours at room temperature, the membranes were imaged on the ChemiDoc Touch imaging system (Bio-rad, USA).

The membrane-associated proteins on CM and CMGCL were detected by Coomassie blue staining and WB. GCL, CM and CMGCL in the loading buffer were first fractionated by SDS-PAGE. For Coomassie blue staining, the gel was washed and then stained using a Coomassie blue fast staining solution, after decolorization in water, a gel imaging system (Tanon, Shanghai, China) was used for gel visualization. For WB analysis, the subsequent procedure was the same as described before.

1. **GSH consumption assay**

The reaction of DTNB and GSH was used to detect the GSH depletion ability of CMGCL. 5 mM GSH solution was mixed with different concentrations of CMGCL with equal volume for 1 hour, the final concentrations of Cu-LDHs were 0, 0.125, 0.25, 0.5, 1 mg/mL, respectively. The GSH concentrations were measured by a GSH content assay kit (Solarbio, Beijing, China) based on the absorbance peak at 415 nm of the generated yellow TNB^2-^.

1. **Detection of glucose and H_2_O_2_**

To verify the catalytic ability of CMGCL, 10 mM glucose solution was mixed with CMGCL (GOx: 50 μg/mL) with equal volumes, the solutions were collected to determine the residual glucose content and H_2_O_2_ content at different time points. In addition, various glucose solutions (0, 1, 5, 10 mM) were mixed with CMGCL (GOx: 50 μg/mL) with equal volumes for 1 hour and the H_2_O_2_ concentrations were measured. The concentrations of glucose or H_2_O_2_ were measured by a glucose or H_2_O_2_ content assay kit (Solarbio, Beijing, China) in terms of the manufacturer’s protocols.

1. **Detection of •OH**

2 μL TMB (0.1 M), 100 μL H_2_O_2_ (1 mM) and 100 μL CMGCL with different concentrations (the final concentrations of Cu-LDHs were 0, 0.25, 0.5, 1, 2 mg/mL, respectively) were reacted for 10 min. TMB, TMB + H_2_O_2_ with the absence of CMGCL were set as controls, and the production of •OH was detected by the TMB absorbance change at 652 nm using UV-vis spectrophotometer (Tecan spark, USA).

1. **Cytotoxicity study**

For CCK-8 assay, LLC cells were cultured in 96-well plates. For low glucose or Es-Cu treatments, normal or low glucose cell medium with or without Es-Cu pulse (Es: 40 nM; CuCl_2_: 10 μM; Pulse for 4 hours) were incubated for a total 24 hours. For nanoparticle treatments, fresh cell medium containing different concentrations of BCL, GCL or CMGCL (0, 0.5, 1, 2, 4, 8, 10 μg/mL) were incubated for another 12 hours, respectively. Then, medium mixed with 10% CCK-8 solutions was replaced to analysis the cell survival rate.

For Live/Dead staining, LLC cells were seeded in 24-well plates, fresh cell medium containing different nanoparticles (4 μg/mL) was replaced. After being cultured for another 12 hours, cells were washed, stained using Calcein AM/PI double stain kit (MKBio, Shanghai, China) and imaged using fluorescent microscopy.

1. **Cell apoptosis and mitochondrial membrane potential analysis**

For cell apoptosis analysis, LLC cells were cultured in 6-well plates overnight, then, they were cultured with BCL, GCL or CMGCL at 4 μg/mL for 4 hours, cells were rinsed and collected for quantitate analysis using Annexin V-FITC/PI cell apoptosis kit (Solarbio, Beijing, China) with a flow cytometer (Beckman CytoFLEX, USA). For membrane potential analysis, LLC cells were cultured on 12-well plates for 24 hours and treated with different nanoparticles as described above, cells were then incubated with JC-1 staining solutions using a JC-1 staining kit (Beyotime, Shanghai, China) to observe the JC-1 aggregates (red) and monomers (green) with fluorescence microscopy. Additionally, cells were also lysed with RIPA buffer for WB to detect the expression change of Bcl2/Bax protein.

1. **Intracellular ROS and GSH detection**

Intracellular ROS levels were measured using flow cytometry with DCFH-DA probe. In brief, LLC cells were cultured with 6-well plates. For low-glc or Es-Cu treatments, normal or low glucose cell medium with or without Es-Cu pulse were incubated for another 24 hours. For nanoparticle treatments, fresh cell medium with BCL, GCL or CMGCL (4 μg/mL) were incubated for another 4 hours. Then, cells were incubated with DCFH-DA probe (10 μM) for 30 min at cell incubator. Cells were collected and washed with PBS for flow cytometry analysis.

For intracellular GSH detection, LLC cells were seeded in 6 cm dishes. Then, they were treated with BCL, GCL or CMGCL at 4 μg/mL, respectively. After 4 hours, cells were collected and the intracellular GSH levels were measured with GSH content assay kit on the basis of the manufacturer’s instruction. The final GSH levels (μg/mg prot) were determined by calculating with the correspondent protein content.

1. **Detection of intracellular cuproptosis protein expression**

The expression changes of cuproptosis pathway-related protein FDX1, LIAS and DLAT were examined to verify cell cuproptosis. LLC cells treated with Es-Cu pulse or different nanomaterials at 4 μg/mL for 4 hours were collected in ice-cold RIPA lysis buffer for further WB analysis of FDX1 and LIAS expression. For DLAT WB analysis, cells were collected in ice-cold NP-40 lysis buffer. Each protein sample was separated by SDS-PAGE at pre-cast Bis-Tris 4-12% gels in MOPS Running Buffer (ACE Biotech., China) and then transferred onto PVDF membranes for further WB analysis as described before.

For observation of DLAT expression, LLC cells were seeded into glass-petri dishes overnight. After being treated with Es-Cu pulse or different nanomaterials at 4 μg/mL for 4 hours, cells were first stained with preheated MitoTracker Deep Red (250 nM) at 37 °C for 30 min. After being fixed with 4% paraformaldehyde (PFA) for 15 min and blocked for 20 min, cells were incubated with anti-DLAT antibody overnight at 4 °C and secondary antibody for 1 hours at room temperature. Finally, cells were washed with PBST and stained with DAPI. Pictures were obtained by confocal laser scanning microscopy (CLSM, Olympus SpinSR, Japan).

1. **PD-L1 expression after different treatments**

To measure the expression of PD-L1, LLC cells were cultured in 6-well plates. For low-glc or Es-Cu treatments, normal or low-glucose cell medium with or without Es-Cu pulse were incubated for another 24 hours. For nanoparticle treatments, fresh cell medium with BCL, GCL or CMGCL (4 μg/mL) were treated for another 4 hours. Cells were harvest and washed with PBS, then stained with PD-L1 antibody for 20 min. After rinsed with PBS, the expression levels of PD-L1 in different cell samples were analyzed using flow cytometry. Besides, the PD-L1 protein levels in LLC cells after treated with nanoparticles were also examined by WB.

1. **Cellular uptake assay**

CLSM and flow cytometry assays were used to determine the cellular uptake efficiency of LLC cells. Cu-LDHs were labeled with FITC, LLC cells were cultured in glass-bottom petri dishes or 12-well plates. Fresh cell culture medium suspended with 5 μg/mL FITC-labeled BCL or CMBCL were replaced and incubated for another 4 hours, cells were washed with PBS three times. The cell nuclei were incubated with Hoechst 33342 for 10 min before photographed with CLSM. Cells were collected and quantified with flow cytometry.

1. ***In vivo* biodistribution and targeting study**

To obtain Cy7 labeled nanoparticles, the BSA solution was first mixed with BSA^Cy7^ at the molar ratio of 9:1,^[3]^ the subsequent procedure was the same as described above. LLC cells were injected into C57BL/6J mice subcutaneously (2 x10^6^ cells per mouse). Mice randomly divided into three groups were intravenously administrated with equivalent free BSA^Cy7^, BCL^Cy7^ and CMBCL^Cy7^ (100 μL in PBS, 4 mg/mL), respectively. Then, living images of mice were photographed with isoflurane anesthesia using Photon Imager Optima (Biospace, France) at different time intervals (1, 6, 12, 24 hours). Mice were sacrificed 24 hours after administration, the tumors and major organs (heart, liver, spleen, lung and kidneys) were harvested for Cy7 fluorescence imaging. M3 Vision analysis software (Biospace, France) was used for subsequent visualization and quantification.

1. ***In vivo* anti-tumor effect and biosafety assays**

C57BL/6J mice were injected with LLC cells subcutaneously (2 x10^6^ cells per mouse). When subcutaneous tumors grew to 50-100 mm^3^, mice were divided into four groups on day 0 randomly. PBS, BCL, GCL, CMGCL (100 μL in PBS, 4 mg/mL) were injected via the tail veins on day 0, 3, 6, 9. The tumor volume and body weight of every mouse were recorded every two days. The tumor volume was calculated using the formula: volume (mm^3^) = longest diameter × shortest diameter^2^/2.

On day 14, mice were sacrificed, and the final tumor weight was recorded. Serum of mice were collected for the serum biochemistry measurement including AST, ALT, BUN, CR, LDH and CK. Tumors and major organs (heart, liver, spleen, lung and kidney) were collected, fixed in 4% PFA and then embedded to obtain tissue sections. Tumor and major organ sections were prepared routinely in process and then subjected to H&E staining. Tumor sections were further analyzed using Ki-67 immunohistochemistry staining, TUNEL assay and DLAT immunofluorescence staining. Images were obtained through microscopy and analyzed using ImageJ software.

For flow cytometry analysis, tumor tissues were excised from sacrificed mice on day 10. Then, tumors were cut into pieces and then digested in a shaker at 37 °C to obtain single-cell suspensions for 45-60 min. Then, cells were filtered and blocked with anti-mouse CD16/32. Cell suspensions were then stained with corresponding fluorochrome-conjugated antibodies for 20 min protected from light. Flow cytometry was performed immediately, and the results were analyzed using FlowJo software.

1. **Combinational therapy via CMGCL and αPD-L1**

Subcutaneous LLC tumor-bearing mice models were established as described above. On day 0, mice were divided into four groups randomly: PBS, αPD-L1, CMGCL and αPD-L1 + CMGCL. CMGCL (100 μL in PBS, 4 mg/mL) were injected via the tail veins on day 0, 3, 6, 9, and αPD-L1 antibody (10 mg/kg) was injected intraperitoneally on day 1, 4, 7, 10. The tumor volume and body weight of every mouse were recorded every two days. The final tumor weight was recorded after mice were sacrificed on day 14. Tumors were dissected, fixed and then embedded in paraffin for further CD8, NK1.1 immunofluorescent staining, respectively. Images were obtained through fluorescent microscopy and analyzed using ImageJ software.

To establish the lung metastasis tumor model, LLC cells were intravenously injected into C57BL/6J mice (1 x 10^6^ cells per mouse). After 5 days, mice were randomly grouped into four: PBS, αPD-L1, CMGCL and αPD-L1 + CMGCL. Treatments were applied the same as above. Mice were sacrificed after 14 days. Lungs were dissected, weighted and subsequently fixed for H&E assay to examine the tumor nodules histologically.

1. **Statistical analysis**

Data were depicted as mean ± SEM from at least three independent experiments. Statistical analyses were performed by GraphPad Prism or Origin software. Comparison between two groups were analyzed using unpaired Student’s t-test while one-way or two-way ANOVA was used for multiple comparisons. Results were considered statistically significant if the *P* values were less than 0.05 (**P* < 0.05, ***P* < 0.01, or ****P* < 0.001).

**Table S1. Antibodies for WB, immunofluorescence or immunohistochemistry**

| **Antibody** | **Resource** |
| --- | --- |
| Anti-GAPDH | HUABIO |
| Anti-Na^+^/K^+^-ATPase | Santa Cruz Biotechnology |
| Anti-N-Cadherin | Cell Signaling Technology |
| Anti-E-Cadherin | Cell Signaling Technology |
| Anti-EpCAM | Abcam |
| Anti-β-actin | Proteintech |
| Anti-Bcl2 | Cell Signaling Technology |
| Anti-Bax | Cell Signaling Technology |
| Anti-LIAS | Proteintech |
| Anti-FDX1 | Abcam |
| Anti-DLAT | Proteintech |
| Anti-PD-L1 | Abmart |
| Anti-Ki-67 | Abcam |
| Anti-CD8a | Abcam |
| Anti-NK1.1 | Abcam |
| HRP-conjugated goat anti-rabbit/mouse secondary antibody | HUABIO |
| Alexa Fluor 488 goat anti-rabbit IgG secondary antibody | Invitrogen |

**Table S2. Sequences of primers**

| **Primer** | **Forward (5’ to 3’)** | **Reverse (5’ to 3’)** |
| --- | --- | --- |
| β-actin | GGCTGTATTCCCCTCCATCG | CCAGTTGGTAACAATGCCATGT |
| Hk2 | ATGATCGCCTGCTTATTCACG | CGCCTAGAAATCTCCAGAAGGG |
| Eno2 | GTCCCTGGCCGTGTGTAAG | CATCCCGAAAGCTCTCAGC |
| Slc31a2 | TAGCAGCCGCAATCCTAGT | GGAGCAAGACTACCAGCACT |
| Slc25a3 | GGCTCCATGAAGTATTATGCACT | AAACCACGAACGCCATCTTCT |
| Aoc2 | CGGTGCATACACACGCTTTC | AGGCATTGGTCTGGTTGGTAG |
| Sod3 | CCTTCTTGTTCTACGGCTTGC | GCGTGTCGCCTATCTTCTCAA |


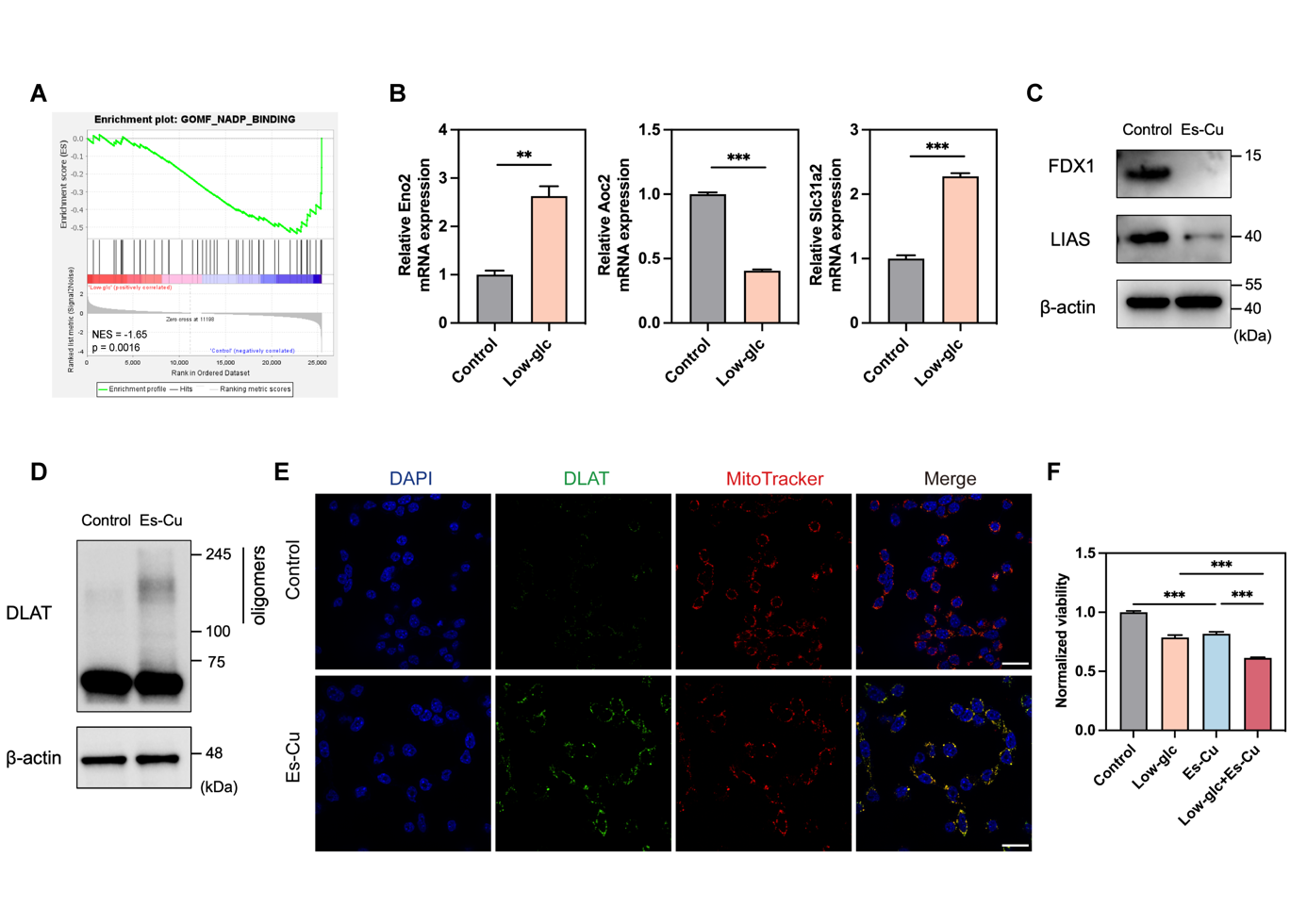


**Figure S1.** (A) GSEA analysis of NADP binding pathway in control and low-glc groups. (B) The relative mRNA levels of Eno2, Slc31a2 and Aoc2 in different groups detected by RT-qPCR and were normalized by the levels of β-actin. (C) WB analysis of FDX1 and LIAS protein expression in control and Es-Cu groups. (D) WB analysis for DLAT and its oligomers of LLC cells after indicated treatments. (E) Representative CLSM photographs of DLAT (green) expression co-stained with MitoTraker (red) and DAPI (blue) in LLC cells with various treatments. Scale bar: 20 μm. (F) Relative cell viability of LLC cells after treated with low-glc or Es-Cu. Data are depicted in mean ± SEM; n=3. ***P* < 0.01, ****P* < 0.001.


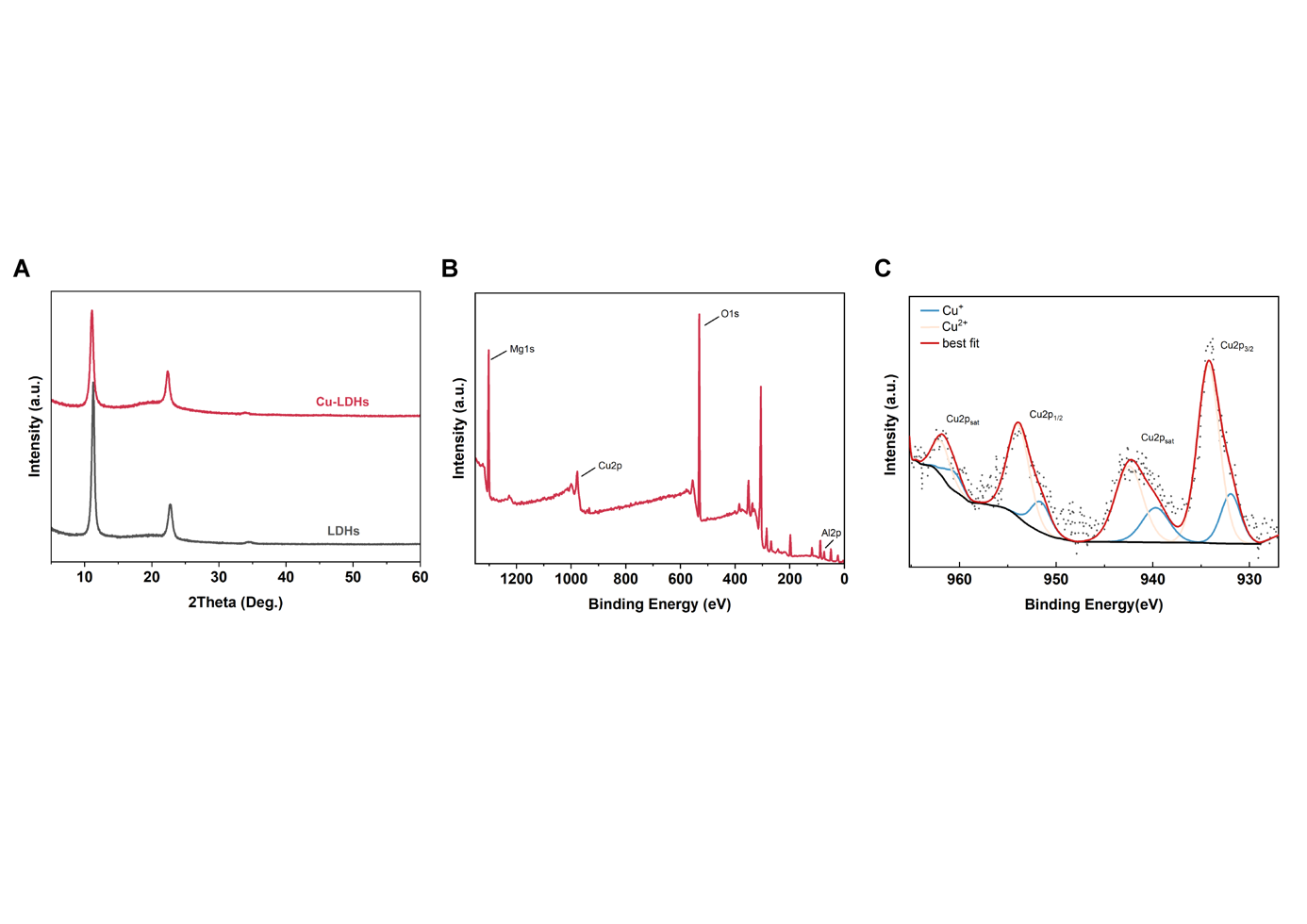


**Figure S2.** (A) XRD patterns of LDHs and Cu-LDHs. (B and C) XPS spectra survey and Cu2p analysis of Cu-LDHs.


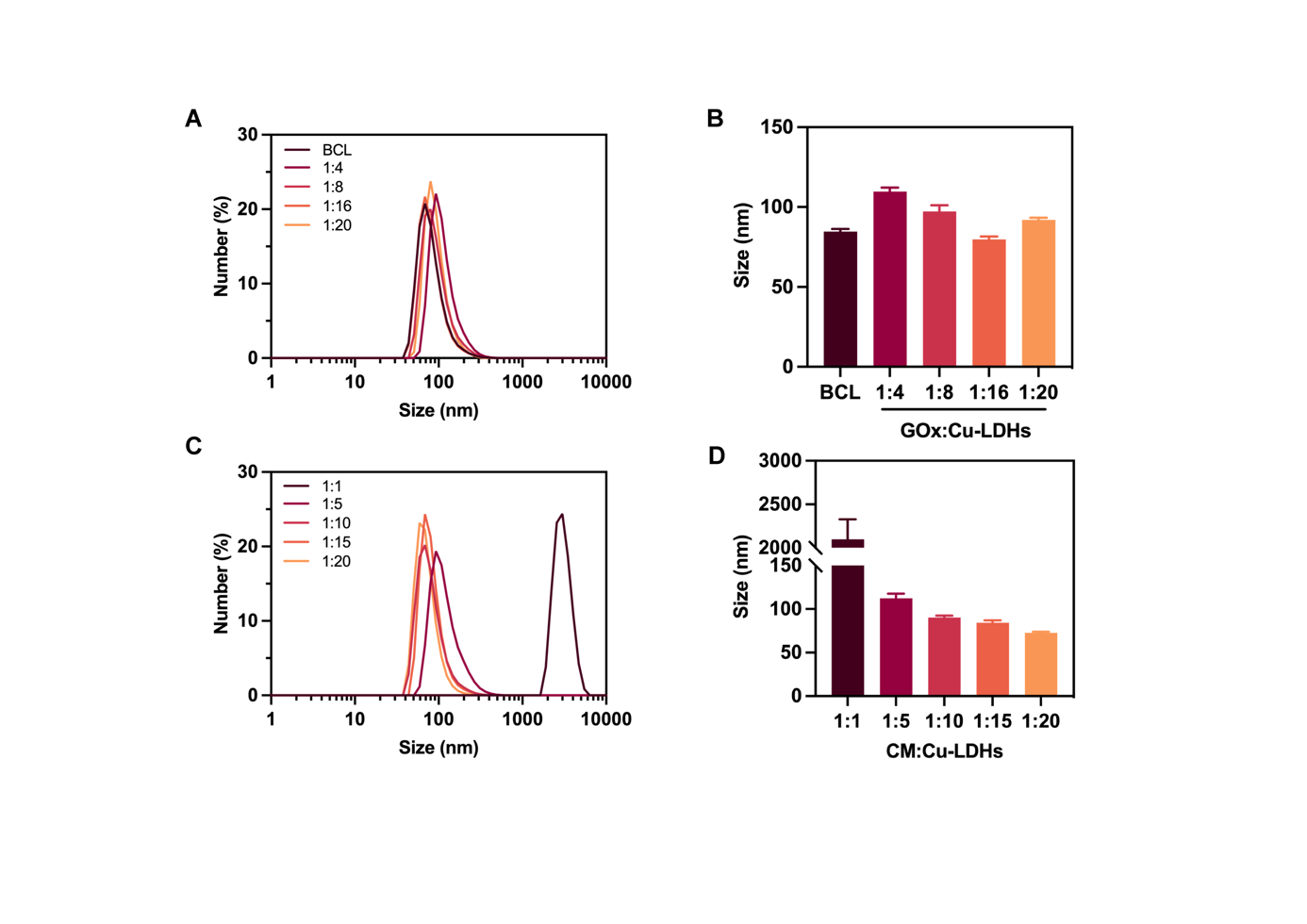


**Figure S3.** (A and B) Particle size distributions and average sizes of BCL and GCL with different mass ratio of GOx and Cu-LDHs, measured by DLS. (C and D) Particle size distributions and average sizes of CMGCL with different mass ratio of CM and Cu-LDHs, measured by DLS. Data are depicted in mean ± SEM; n=3.


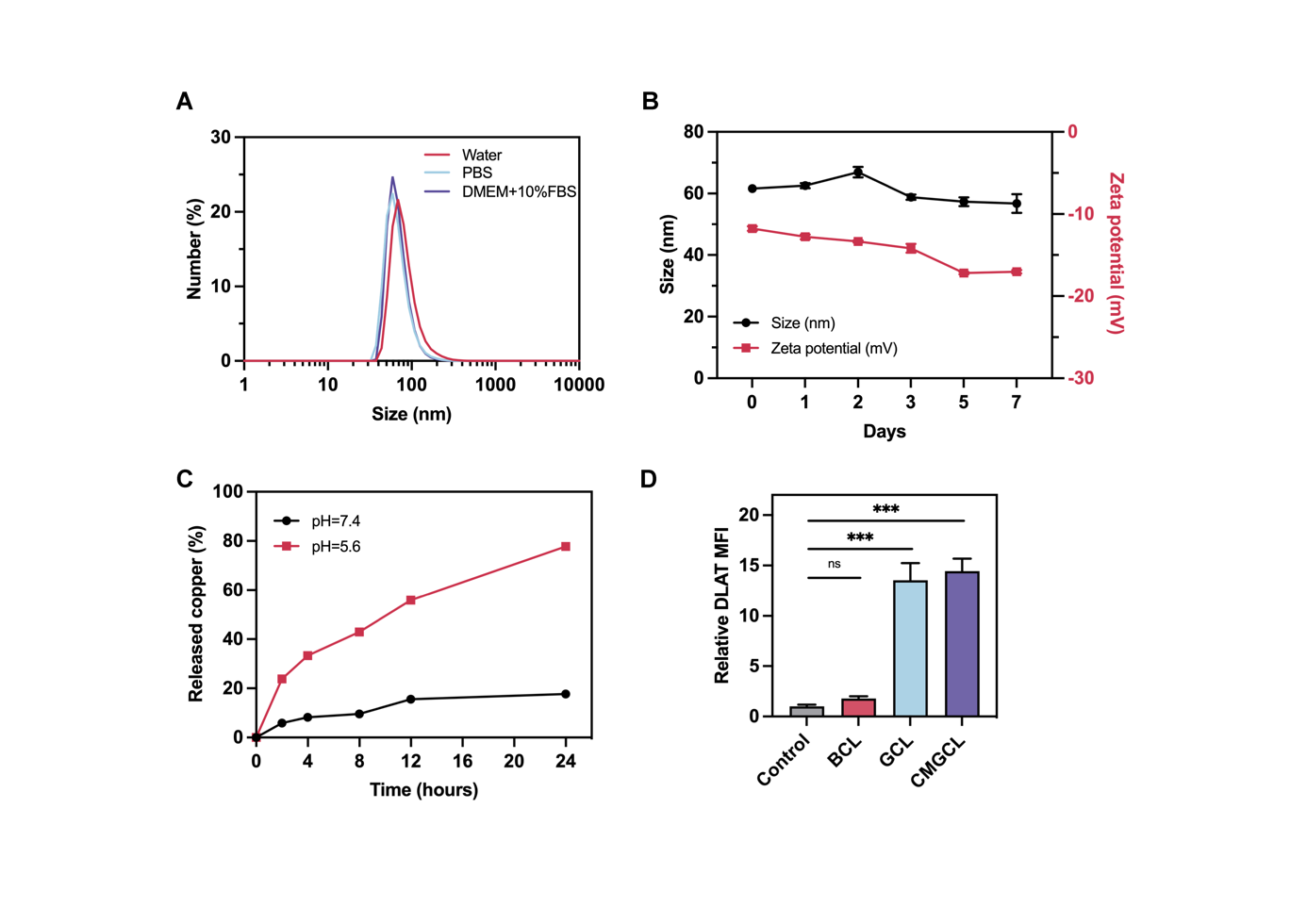


**Figure S4.** (A) Particle size distributions of GCL in deionized water, PBS or DMEM + 10% FBS for 24 hours. (B) Average size and zeta potential of GCL in PBS for 7 days. (C) Release curves of copper ions under PBS with different pH values (pH=7.4 or pH=5.6). (D) Relative quantitative analysis of DLAT fluorescence intensity in each group. MFI: mean fluorescence intensity. Data are depicted in mean ± SEM; n=3. ns, not significant, ****P* < 0.001.


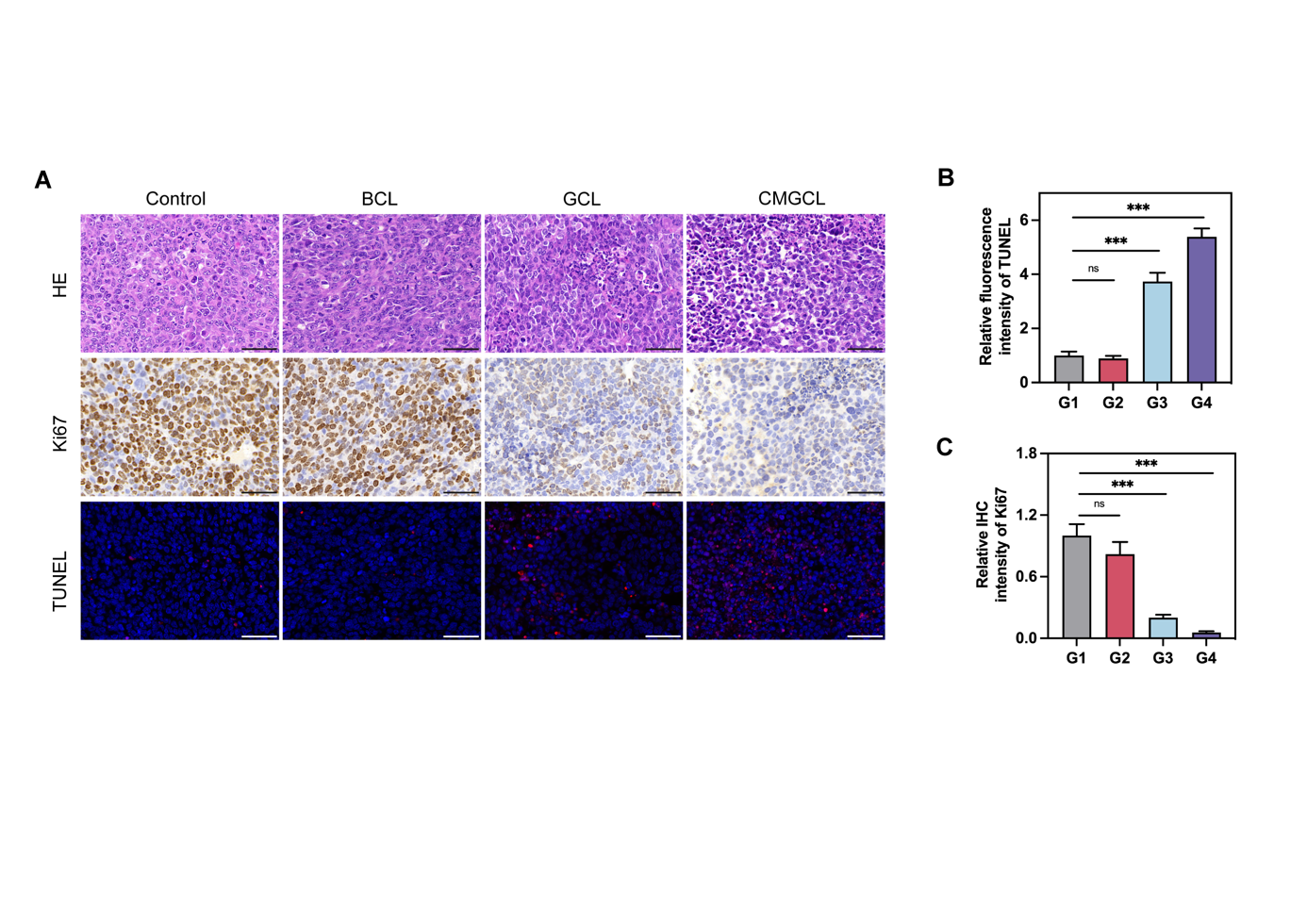


**Figure S5.** (A) Representative H&E, Ki-67 and TUNEL images of tumor sections 14 days in different groups. Scale bar: 50 μm. (B and C) Corresponding relative fluorescence intensity of Ki-67 and TUNEL after different treatments. G1, Control. G2, BCL. G3, GCL. G4, CMGCL. Data are depicted in mean ± SEM; n=3. ns, not significant, ****P* < 0.001.


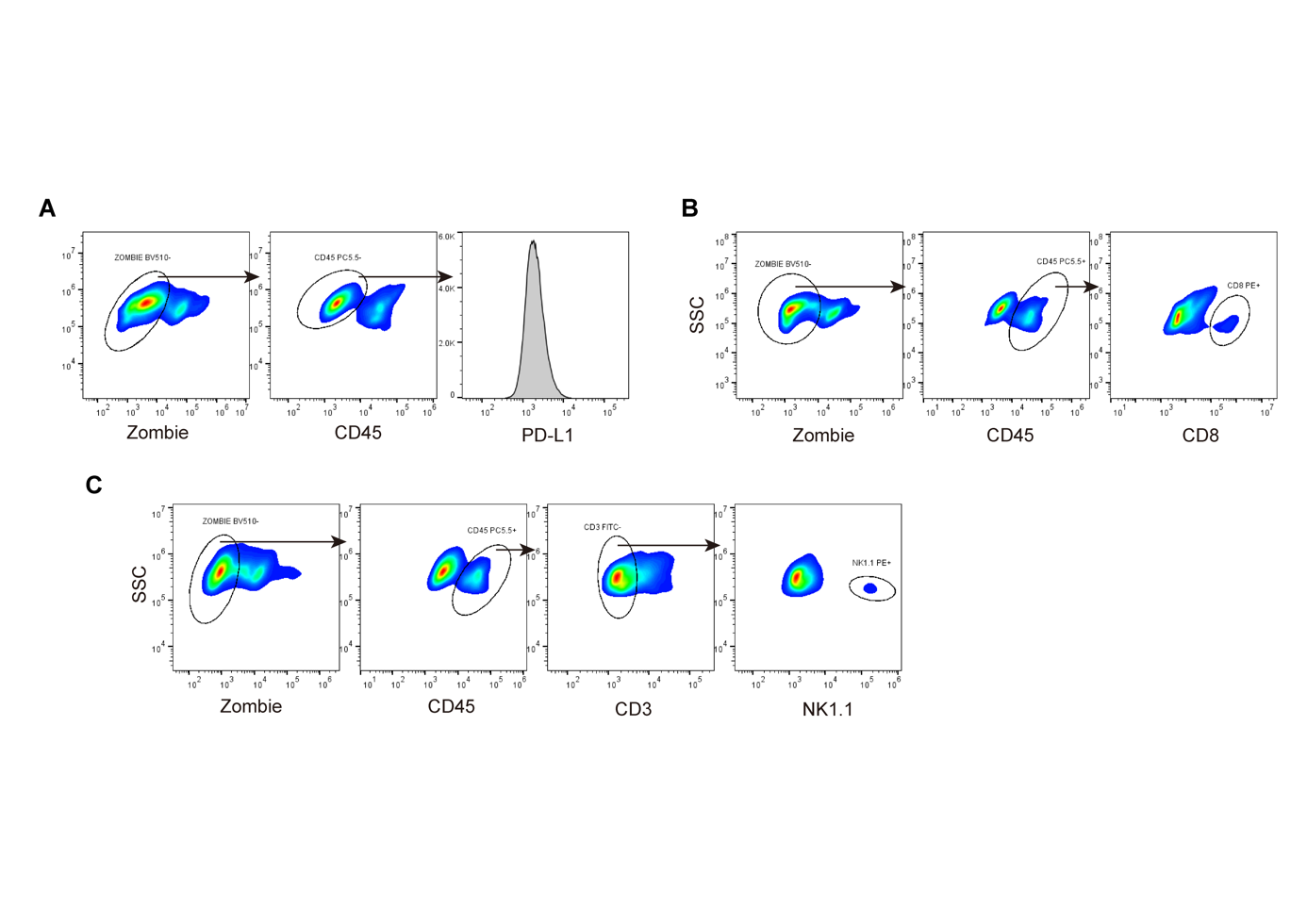


**Figure S6.** (A to C) Flow cytometry gating strategies for experiments in Fig. 7H, Fig. 7J and Fig. 7L.


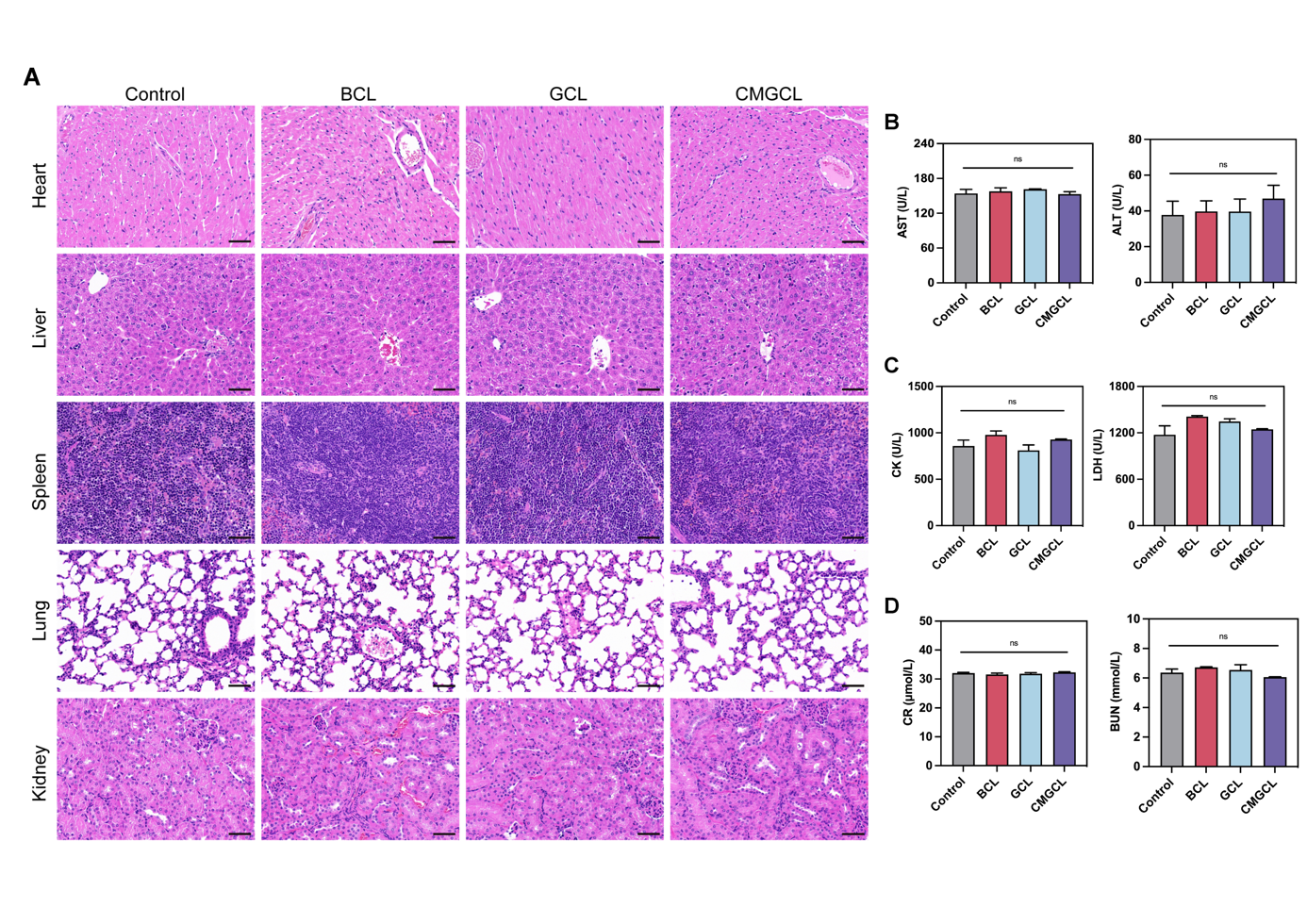


**Figure S7.** (A) Representative H&E pictures of hearts, livers, spleens, lungs and kidneys of mice in different groups on day 14. Scale bar: 50 μm. (B to D) Biochemical indicators including AST, ALT, CK, LDH, CR, BUN of the serum for mice with various treatments. Data are depicted in mean ± SEM; n=3. ns, not significant.


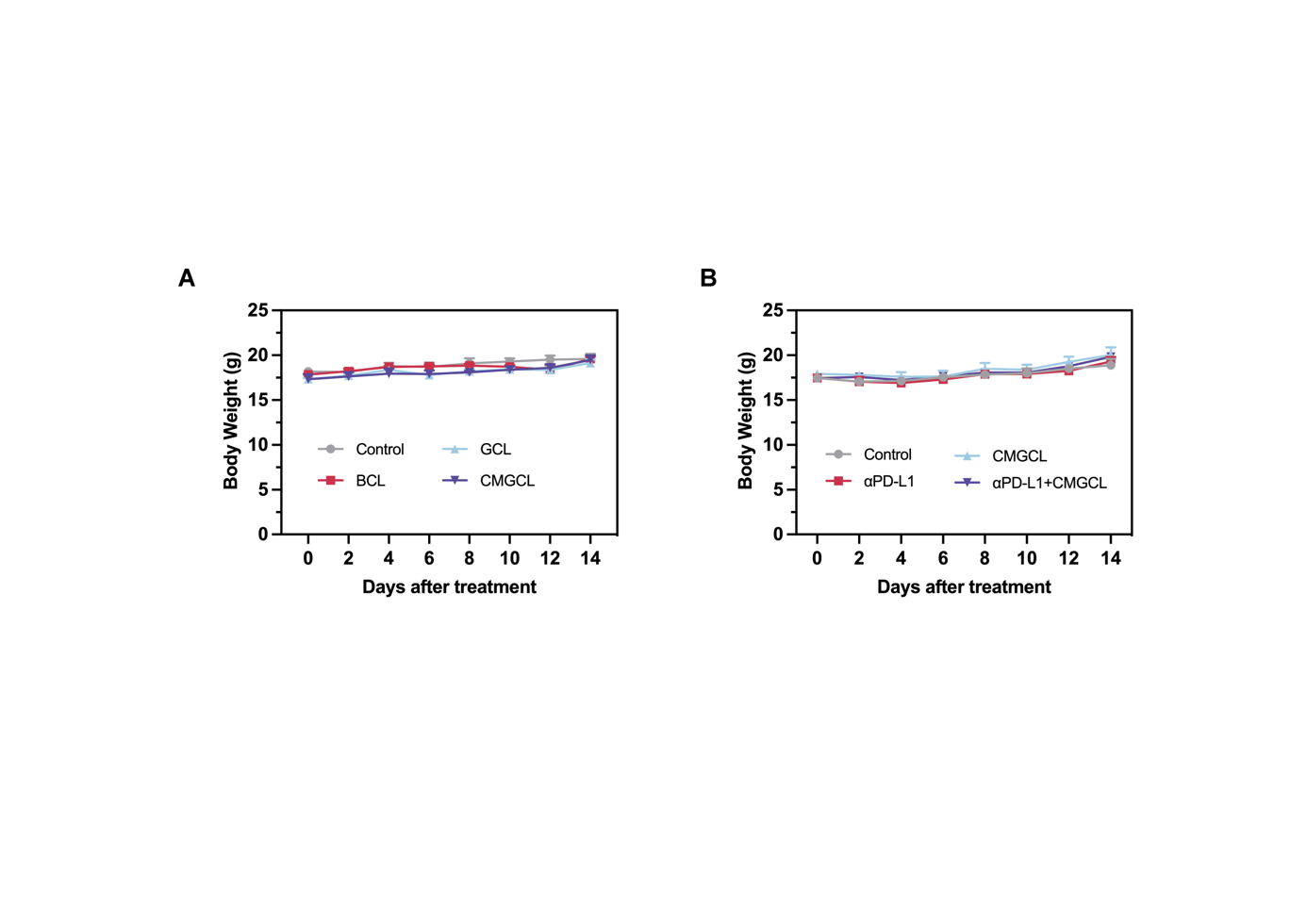


**Figure S8.** (A and B) Body weight changes of LLC tumor-bearing mice post different treatments. Data are depicted in mean ± SEM; n=5.

**References**

[1] a) C.-N. Members, Partners, Nucleic Acids Res 2022, 50 (D1), D27, https://doi.org/10.1093/nar/gkab951; b) T. Chen, X. Chen, S. Zhang, J. Zhu, B. Tang, A. Wang, L. Dong, Z. Zhang, C. Yu, Y. Sun, L. Chi, H. Chen, S. Zhai, Y. Sun, L. Lan, X. Zhang, J. Xiao, Y. Bao, Y. Wang, Z. Zhang, W. Zhao, Genomics Proteomics Bioinformatics 2021, 19 (4), 578, https://doi.org/10.1016/j.gpb.2021.08.001.

[2] a) C. Gao, Q. Huang, C. Liu, C. H. T. Kwong, L. Yue, J. B. Wan, S. M. Y. Lee, R. Wang, *Nat Commun* **2020**, *11* (1), 2622, https://doi.org/10.1038/s41467-020-16439-7; b) Q. Wang, H. Wang, H. Yan, H. Tian, Y. Wang, W. Yu, Z. Dai, P. Chen, Z. Liu, R. Tang, C. Jiang, S. Fan, X. Liu, X. Lin, *Sci Adv* **2022**, *8* (26), eabn3333, https://doi.org/10.1126/sciadv.abn3333.

[3] W. Chen, H. Zuo, E. Zhang, L. Li, P. Henrich-Noack, H. Cooper, Y. Qian, Z. P. Xu, *ACS Appl Mater Interfaces* **2018**, *10* (24), 20326, https://doi.org/10.1021/acsami.8b04613.
